# Utilizing strategy mapping software for participatory evaluation of crisis management efforts: A case study

**DOI:** 10.1371/journal.pone.0356419

**Published:** 2026-08-20

**Authors:** Anton Björnqvist, Carl-Oscar Jonson, Erik Prytz, Björn J. E. Johansson, Peter Berggren

**Affiliations:** 1 Center for Disaster Medicine and Traumatology, Region Östergötland, Linköping, Sweden; 2 Department of Computer and Information Science, Linköping University, Linköping, Sweden; 3 Department of Biomedical and Clinical Sciences, Linköping University, Linköping, Sweden; Public Library of Science, UNITED KINGDOM OF GREAT BRITAIN AND NORTHERN IRELAND

## Abstract

This multi-stage study explores the novel application of the strategy mapping methodology, incorporated into a software tool, to conduct participatory evaluations of crisis management efforts. The methodology was selected because it has shown promise in other fields of research, aligns with participatory evaluation principles, and addresses the need for more impactful evaluations of crisis management efforts. The evaluation was conducted through a four-step workshop series with 15 healthcare professionals, which culminated in ten participant-developed recommendations. The recommendations targeted central risks as determined throughout the workshop series using the strategy mapping software. Approximately one year after the workshop series, semi-structured interviews with ten of the workshop participants were conducted to assess the perceived use and value of the recommendations and the evaluation process. The findings indicate that the evaluation was impactful, suggesting that the strategy mapping methodology is a viable approach for conducting participatory evaluations of crisis management efforts. Insights are also provided regarding how future participatory evaluations of crisis management efforts can be improved. These insights include the potential need for increased involvement of the evaluator in producing recommendations to secure their clarity and actionability. Furthermore, it is argued that aspects of the strategy mapping methodology perceived as valuable, such as participants rating recommendations, should be incorporated into future participatory evaluations of crisis management efforts even if the complete strategy mapping methodology is not applicable or available. By applying findings from this study, the impact of evaluations of crisis management efforts can be increased, thereby contributing to improved preparedness for future crises.

## Introduction

Evaluations of crisis management efforts (i.e., crisis exercises and crisis responses) are often criticized for having limited impact (i.e., their findings are rarely used in practice) and for not providing novel insights into how to strengthen crisis preparedness [1,2]. Potential explanations for this, based on insights from similar fields of research, concern how evaluations are conducted and how results are presented [3–6]. It has also been shown that aspects related to the potential impact of evaluations of crisis management efforts are seldom mentioned in the existing literature [7]. This lack of impact, and a general neglect of the impact of evaluations of crisis management efforts, have negative consequences since evaluations of crisis management efforts hold great potential when aiming to learn from experiences and for strengthening crisis preparedness [8–11]. There is therefore a need for approaches emphasizing the impact of evaluations of crisis management efforts, and research into such approaches, to increase the likelihood of such evaluations contributing to strengthening crisis preparedness [2,12]. Such research would go beyond the scope of much of the available research on evaluations of crisis management efforts, which primarily focuses on developing and describing methods and assessment criteria for assessing crisis management processes and performance [7,13–15].

Previous research shows tendencies that stakeholder involvement in evaluations of crisis management efforts can increase evaluations’ direct impact [2]. Such approaches might therefore prove useful in the crisis management context when aiming to conduct impactful evaluations. A specific approach incorporating and emphasizing the advantages of stakeholder involvement is participatory evaluation [16]. Central to the participatory evaluation approach is, as the name implies, the active involvement of relevant and affected stakeholders in the evaluation process [17]. However, the approach does not prescribe specific methods for how to evaluate or involve stakeholders [18]. This implies that methods from adjacent fields of research emphasizing stakeholder involvement could be used as inspiration when determining how to conduct participatory evaluations. One such method, developed across different fields such as public management and operations research, is strategy mapping [19,20]. Originally developed to foster strategy development in organizations, strategy mapping makes use of stakeholders’ experiences and knowledge to develop strategies for how to achieve set objectives [21]. Due to the high degree of stakeholder involvement in strategy mapping, and its promising results for developing actionable strategies suited for implementation [19,22], it is of interest to examine how it can be used as a method for participatory evaluations of crisis management efforts. Furthermore, recent calls within the participatory evaluation literature emphasize the need for longitudinal case studies that examine participatory evaluation applications and their subsequent evaluation consequences over time, through the perspectives of stakeholders actively engaged in these evaluations [23].

Employing the strategy mapping method for participatory evaluation adheres to suggestions that mapping methodologies are suitable for participatory evaluation [24]. It also adheres to calls stating that participatory evaluation methods must be culturally sound [25], as evident by the fact that the strategy mapping methodology has previously been employed in addressing complex and crisis-like contexts [26,27], although in non-evaluative inquiries. Furthermore, although similar methods for participatory evaluation exist, such as ripple effects mapping [28], strategy mapping, as employed in this study, differs due to its increased focus on the mapping procedures and on producing actionable recommendations based on experiences from a crisis management effort, rather than focusing on determining the impact of interventions.

The often-limited impact of evaluations of crisis management efforts is therefore addressed in this case study by exploring the application of the strategy mapping methodology, incorporated into a software tool, as a method for participatory evaluation. This includes examining how the methodology can be used to conduct participatory evaluations, and how the evaluation process and its outcomes are perceived and used by stakeholders involved in, and affected by, the evaluation. The case study represents a novel application of such a tool in the context of evaluations of crisis management efforts. The study is guided by the following research questions:

- How can the strategy mapping methodology, incorporated into a software tool, be used to conduct participatory evaluations of crisis management efforts?
- How are the evaluation process and its recommendations used by the evaluated organization and perceived by stakeholders involved in, and affected by, the evaluation?

In addition to the research questions, the study also aims to inform future evaluations on how to effectively involve stakeholders and how to incorporate relevant aspects of the strategy mapping methodology. By answering the research questions and fulfilling the study’s aims, this study contributes to a better understanding of how the impact of evaluations of crisis management efforts can be increased. By increasing the impact of such evaluations, greater opportunities for learning from past experiences can be created, which in turn can increase preparedness for future crises.

To answer the two research questions, a suitable past crisis management effort had to be selected to be evaluated and serve as a case. For this study, the COVID-19 pandemic was selected. The pandemic was selected due to its extended duration, being classified as a public health emergency of international concern from 2020 to 2023 [29]. This duration suggests that multiple areas needing improvement can be identified through evaluation, as well as opportunities to develop recommendations targeting identified areas. Furthermore, the length of the pandemic also suggests that multiple stakeholders were involved in the crisis management efforts, indicating the availability of diverse perspectives. It is, however, important to note that this study does not aim to contribute to knowledge or lessons learned about or from the pandemic. Rather, the pandemic is used as an instrumental case [30] and as a context to explore the research questions (i.e., how the strategy mapping methodology can be used to conduct participatory evaluations of management efforts).

## Theoretical background

Participatory evaluation and strategy mapping are the two main approaches underpinning this study. In this section, they are introduced and described under separate headings to support understanding of the subsequent parts of the article.

### Participatory evaluation

Participatory evaluation evolved from evaluation approaches emphasizing stakeholder involvement. The main reasoning behind the development of participatory evaluation was that increasing the involvement of stakeholders in evaluations ought to enhance evaluation use [16]. Although often not defined in further detail in terms of exact methods, the main element of participatory evaluation is that evaluators must collaborate with and involve stakeholders from the evaluated entities before, during, and after evaluations [31,32]. The degree of involvement and the justifications for why stakeholders should be involved in the evaluation process depends on the form of the participatory evaluation approach [18,33]. However, it has been stated that for an evaluation to adhere to the participatory approach, the stakeholders should be actively involved in conducting the evaluation [34]. Furthermore, participatory evaluation is theoretically supported by, and to some degree grounded in, theories of organizational learning, which emphasize that collaborative approaches foster shared understanding and ownership to support organizational improvement [16,35].

According to Weaver and Cousins [33], there are primarily three justifications for using a participative evaluation approach. The first justification is pragmatic, meaning that stakeholder involvement is justified based on the assumption that it will increase the evaluation’s impact. The second justification is political, meaning that the involvement of stakeholders is justified based on the assumption that such involvement will provide marginalized groups with an opportunity to voice their opinions. The third justification is epistemological, meaning that involvement of stakeholders is justified based on the assumption that a participatory approach is the optimal approach for producing valid knowledge of social phenomena. These justifications are closely linked to the various forms of participatory evaluation as each form highlights approaches for how to fulfil the justifications. One of the most used forms, closely connected to the pragmatic justification, is practical participatory evaluation, based on the assumption that the involvement of stakeholders will result in an impactful evaluation [18]. According to Greene [36], the increased impact from participatory evaluations might stem from participants acquiring an increased understanding and ownership of evaluation results, while also contributing to increased perceptions of the conclusions as reliable and obligations to make use of the conclusions. Furthermore, beyond the potential to increase an evaluation’s impact through the use of recommendations or other findings, it has been suggested that the participatory evaluation process itself can be impactful as stakeholder involvement provides an opportunity for reflection and learning among participants throughout the evaluation process [25,37]. These positive aspects of participatory approaches for evaluations have also increased interest in potential applications within fields like crisis management [28].

After identifying why stakeholders should be included in the evaluation, as emphasized in justification and form, the next steps relate to which degree they should be included and how they should be included (i.e., the methods of the evaluation). According to Daigneault and Jacob [38], there are primarily four tasks where stakeholders can be involved. The first task is formulating evaluation questions and methodological design, emphasizing that stakeholders can be included in various parts of framing the evaluation in terms of evaluation purpose, targeted issues, theoretical frameworks, and methodological design. The second task is data collection and analysis, highlighting that stakeholders can be involved in deciding how to collect data, actively partaking in the data collection or analyzing data. The third task is judgments and recommendations formulation, emphasizing that stakeholders can be involved in judging the evaluated entity or providing recommendations for how to develop the evaluated entity. The fourth task is report and dissemination of evaluation findings, highlighting that stakeholders can be involved in writing and disseminating evaluation findings. To be categorized as a participatory evaluation, a diverse set of stakeholders should be involved in at least one of these aforementioned tasks and possess some control of the evaluation process.

What becomes clear, then, is that there are no particular methods for how to evaluate and involve stakeholders in participatory approaches. Rather, what makes an evaluation participatory is whether stakeholders are actively involved in the evaluation and whether the evaluation adheres to the principles of participatory evaluation in terms of purpose and justification [24]. Burke [24] does, however, propose a set of methods suited for participatory evaluation, which includes group mapping, workshops, and web charts to trace root causes for or effects of specific phenomenon. It is also emphasized that visual and immersive methods of data collection are suitable.

Although participatory evaluation has been praised for providing results that are valuable and likely to be used, there are some criticisms of the approach. The main criticism lies in that the participatory approach to evaluation negatively affects evaluation objectivity, that it is difficult to organize and include all relevant stakeholders, and that it is difficult to determine the impact of including stakeholders [37]. However, Johnson et al.‘s [4] and Searle et al.’s [6] reviews of evaluation use do provide empirical support for the importance of involving stakeholders in evaluations when aiming to conduct impactful evaluations.

### Strategy mapping

The roots of strategy mapping can be traced to methods of cognitive mapping in psychology. Initially named cognitive mapping as the maps were presented as a way of representing thoughts through causal links, the method was later renamed to causal mapping. The name change was done to avoid implicating that the maps represented the mind and due to the method spreading to and being updated within other fields of research [39]. Central to the causal mapping process is the creation of a map, by an individual or a group, with statements and links between statements signifying potential causal relationships [40].

As causal mapping became widely used within organizational and management sciences, it has primarily become a method for initial knowledge elicitation (i.e., as a way of accessing individual and group beliefs in applied settings), followed by analyses and conclusions dependent on the purpose for which the mapping was used. In particular, causal mapping techniques making use of direct elicitation procedures have become increasingly popular. In direct elicitation procedures, the mapping process, involving specifying beliefs and causally linking, is centered around participants specifying, usually by writing, aspects to be mapped and causally linked [41].

In addition to being used as a method for knowledge elicitation and representation, causal mapping has been incorporated into problem structuring methods (i.e., methods for creating representations of problems and developing actions for how to resolve identified problems). More specifically, causal mapping is a cornerstone of the problem structuring method strategic options development analysis (SODA) [42,43]. In SODA, causal mapping techniques are used to create representations of how groups perceive problematic situations (i.e., using causal mapping for knowledge elicitation). In the SODA approach, however, causal maps are not seen as the primary output. Instead, the causal maps serve as a tool for creating a shared understanding of the problematic situation, and for developing actions and strategies targeting the most significant problems submitted to the causal maps. Identifying the most significant problems can be done through visually inspecting the causal maps to find, for example, problems seemingly causing many other problems. One of the main advantages of SODA is that the process is believed to facilitate an attachment to the outcomes, due to the extensive stakeholder involvement, something which in turn increases the probability of the outcomes becoming implemented [44].

Influenced by SODA, strategy mapping has been presented as a further development emphasizing the importance of creating actions and strategies that come to be implemented [45]. Similarly to SODA, strategy mapping has been described as a visual causal mapping process allowing groups to increase their knowledge of and generate ideas about what to do, how to do it, and why to do it [19]. Central to the process is the facilitation of groups as they reason, discuss and connect statements with arrows representing causality. It has been theorized that the visual components of the process foster a coherent understanding and collective agreement of issues, goals, and strategies [46]. Bryson et al. [45] divide the strategy mapping process into four subtasks which must be conducted to create actionable strategies. The first subtask is brainstorming statements, meaning that statements (e.g., problems, risks, goals, etc.) should be generated by participants according to an initial question specifying the area of interest. The second subtask is clustering the statements, meaning that the facilitator and participants organize the statements according to topics. The third subtask is causally linking the generated statements following the causal mapping process, resulting in a map with causal links between submitted statements. The fourth, and last, subtask is using the causal map to identify what to do, how to do it, and why to do it. Furthermore, the strategy mapping process has been incorporated into multiple computer applications aiming to assist and facilitate the process. These applications often include interactive mapping solutions and algorithms for quickly and reliably identifying central statements in the causal map, and tools to increase the participatory involvement through, for example, interactive ratings [20,47]. Among these applications is the browser-based software Strategyfinder (hereinafter primarily referred to as the strategy mapping software) [20,48].

In Strategyfinder, the four subtasks of the strategy mapping process are incorporated into a collaborative browser-based software accessed individually by participants through personal computers. Following the four subtasks of the strategy mapping process, under the guidance of a facilitator, participants can enter statements on a digital whiteboard. Entered statements become instantly visible across all participants’ views. The statements can also be freely moved and clustered by the facilitator in collaboration with the participants. It is also possible for the participants to draw causal links between the statements. The strategy mapping software also includes functions such as creating multiple views and interactive ratings. Lastly, it includes several analytical tools to facilitate the process of identifying the most urgent statements. These tools include identifying statement centrality based on the number of out- and in-going causal links, and identifying loops and potent loop statements (i.e., statements continuously reinforcing one another through causality and statements evident in multiple loops) [48].

Varieties of the strategy mapping process have primarily been used in operations research, business and management research, and public management research [20,49,50]. Recently, the process has also started to spread to other fields of research, including crisis and disaster management research where it, for example, has been used to develop strategies for flooding and to identify systemic risks [27,42,51]. Suggestions have also been made to increase the usage of causal mapping in evaluations. The suggestions primarily present causal mapping as a tool for synthesizing data from diverse sources to make causal assertions, rather than incorporating causal mapping into a strategy mapping framework which would include participatory processes for developing recommendations targeting the most central aspects of the causal map [52].

## Methods

The study was conducted as an instrumental case study [30] with a multi-stage qualitative design, divided into two separate but related stages. In the first stage, the strategy mapping methodology was used to evaluate the COVID-19 pandemic. In the second stage, interviews were conducted to explore participants’ perceptions of the evaluation process and to examine whether the resulting recommendations were used. The two stages allowed for in-depth analysis of the evaluation process in terms of its application and subsequent use. The section begins with an in-depth description of the selected case.

### Case description

In Sweden, twenty-one regional public healthcare organizations are responsible for providing healthcare to inhabitants within their respective catchment areas. The regional public healthcare organizations are also responsible for preparing and managing crises affecting healthcare within their catchment areas [53]. One such crisis impacting all regional public healthcare organizations was the COVID-19 pandemic. In particular, the COVID-19 pandemic put pressure on the regional public healthcare organizations’ units for communicable disease and infection control, suggesting that these units were central in the crisis management processes during the pandemic. The COVID-19 pandemic, and these units in particular, therefore provide a suitable context for evaluation, and by extension also a suitable context for testing novel methods for how to evaluate crisis management efforts. For this study, a specific unit for communicable disease and infection control in one of Sweden’s largest regional public healthcare organizations was chosen as the entity to be evaluated and involved in the evaluation process according to participatory evaluation principles. During the pandemic, the selected unit for communicable disease and infection control was placed in the regional public healthcare system’s command and control structure. Tasks performed by the unit included developing guidelines for how to limit the spread of the disease within hospitals, providing recommendations and other measures to limit the spread of the disease among the public, contact tracing, and generally supporting the decision makers in the command and control structure.

### Evaluation method

The evaluation was designed in accordance with principles and assumptions from practical participatory evaluation [18], using the strategy mapping methodology [45] incorporated into the Strategyfinder software (version 1.0.0). Using these two perspectives and methodologies, the evaluation emphasized stakeholder involvement, collective reflection, and actionability, by involving stakeholders in problem formulation, data interpretation, validation, and recommendation development.

Practically, the evaluation was divided into four workshops, each lasting roughly two hours, focusing on improving the unit for communicable disease and infection control’s crisis management processes based on experiences from the COVID-19 pandemic. Three of these workshops were conducted remotely, with participants and the evaluator, acting as a facilitator, being connected via the videotelephony software Zoom while using the strategy mapping software. The participants connected to the strategy mapping software through their own computers with personal log-in details. The fourth workshop was conducted in-person. Throughout the workshops, the participants were guided by the facilitator. However, the facilitator adopted a hands-off approach to minimally influence the findings of the evaluation, meaning that the participants were responsible for the contents and conclusions of the workshops and the evaluation (e.g., producing recommendations) while the evaluator, acting as a facilitator, was responsible for the workshop and evaluation processes (e.g., how recommendations were produced).

All employees of the unit, consisting of fifteen individuals, participated in the evaluation process, although some were absent on certain occasions. The participants included both managers and staff, consisting of nurses and physicians specialized in infection prevention and control.

The first workshop was conducted on the 3^rd^ of April 2023 and started with a blank surface in the strategy mapping software with the question *“What risks do you perceive during future pandemics, or similar events, based on your experiences from the COVID-19 pandemic?”.* To answer this question, a blind gather was conducted, which means that each participant was asked to anonymously provide up to five answers to the question without being able to see the other participants’ answers. While the participants provided answers to the question, the facilitator thematically grouped the answers on the previously blank surface. Next, the participants were asked to read the complete set of answers and add additional answers during an open gather (i.e., the participants could see each other’s answers immediately). When no more answers were being provided, the complete set of answers was discussed by the participants under the guidance of the facilitator with the purpose of letting the participants familiarize themselves with all answers and merge potential duplicates. Next, the participants were asked to causally link the provided answers if they believed risk A could cause risk B, and so on. Participants could link as many risks as they deemed relevant, with links instantly visible to all. When the causal linking was completed, the most central risks were identified by asking the participants if any risks were particularly central and by using the strategy mapping software’s tools to identify particular risks causing multiple other risks. After identifying central risks, the participants were asked to rate the central risks based on importance on a scale of 1–10. These ratings guided the upcoming workshop. Furthermore, the identified risks were not only seen as potential challenges but also represented areas needing improvement to strengthen future preparedness.

The second workshop was conducted on the 6^th^ of April 2023 to validate the risk system (i.e., the risks and the causal links) from the first workshop. To proceed with the validation, some preparatory steps needed to be taken between the first and second workshops. These preparatory steps primarily included creating five subsystems. The subsystems were identified based on risk centrality using the strategy mapping software’s tools. This means that each subsystem contained one central risk and its immediate environment, which were identified based on the following criteria:

- It was prominent in negative loops.
- It was prominent as a driver of multiple risks.
- It was deemed important by the participants during the first workshop.

Once the second workshop began, each subsystem was addressed separately to let the participants discuss the risks and the causal links, and, if needed, change or add risks and causal links. At the end of the second workshop, the participants were asked to rate the subsystems based on importance on a scale of 1–10. The participants’ ratings guided the coming workshop.

The third workshop was conducted on the 24^th^ of April 2023 to develop and assess recommendations targeting the most central risks (i.e., risks involved in negative loops, risks prominent as drivers of multiple risks, and important risks as determined by the participants). Before the third workshop, preparatory steps were taken. These preparatory steps included creating four subsystems. The subsystems were identified by determining the most central risks using the same analyses as before the second workshop, but took the changes made and the participants’ ratings during the second workshop into account. Once the third workshop began, each subsystem was addressed separately. For each subsystem, the participants were asked to freely, through an open gather, provide recommendations and link the recommendations to the risks which the recommendations aimed to mitigate. When no further recommendations were being added, the participants were asked to rate the recommendations according to perceived effectiveness and feasibility. The ratings were conducted by handing the participants seven markers for each of the two categories, followed by letting them distribute these on the recommendations as they pleased. Recommendations deemed effective and feasible were selected to be further assessed during the fourth workshop.

The fourth workshop was held in-person outside the strategy mapping software on the 12^th^ of June 2023, with the purpose of validating the recommendations developed during the third workshop. Before the workshop, the results from the previous workshops were summarized in a brief report sent out to the participants. The report also included the recommendations developed during the third workshop. Once the workshop began, each recommendation was assessed and discussed by the participants, allowing them to suggest necessary changes. Proposed changes were incorporated into the recommendations, resulting in final recommendations presented in the evaluation report, together with the risks which each recommendation aimed to mitigate.

In Table 1, a summary of the workshops conducted between April and June 2023 is presented. Each workshop built upon the outcomes of the previous one, moving from identifying risks to validating and refining recommendations. Table 1 highlights the objective of each workshop, the preparatory work undertaken before each workshop, and the key procedures conducted for each workshop.

**Table 1:** Summary of the workshops conducted.

Workshop	Date	Objective	Preparatory Steps	Key Procedures
1 (Strategy mapping software).	3 April 2023.	Identify perceived risks during future pandemics based on COVID-19 experiences.	None (started with blank strategy mapping surface).	• Guiding question introduced. • Blind gather. • Facilitator grouped answers. • Open gather. • Discussion to merge duplicates. • Causal linking of risks. • Identified central risks using the strategy mapping software’s tools and participant input. • Rated central risks.
2 (Strategy mapping software).	6 April 2023.	Validate risk system and refine causal links.	Created five subsystems. based on risk centrality.	• Discussed each subsystem separately. • Modified and added risks and links. • Rated subsystems.
3 (Strategy mapping software).	24 April 2023.	Develop and assess recommendations targeting most central risks.	Created four subsystems based on updated analysis of centrality and previous ratings.	• Open gather for recommendations. • Linked recommendations to risks. • Rated recommendations for effectiveness and feasibility. • Selected feasible and effective recommendations for next workshop.
4 (In person).	12 June 2023.	Validate and finalize recommendations.	Summarized previous results and recommendations in a report sent to participants.	• Discussed and assessed each recommendation. • Decided on final recommendations.

### Interviews

During the spring of 2024, approximately one year after conducting the last evaluation workshop, the 15 participants from the evaluation process were asked to be interviewed in a follow-up study. Of these, ten expressed interest in participating, all of whom were still employed at the same unit at the time. As a result, ten semi-structured interviews were conducted. Each interview was conducted for roughly one hour using the videotelephony software Zoom. The interviews were conducted in Swedish by one of the authors, who had also facilitated the evaluation process. The built-in recording feature in Zoom was utilized to record the interviews.

The interviews followed an interview guide divided into three separate parts. For the first part, each recommendation from the evaluation was addressed separately. During this part, the interviewees were asked whether each recommendation had been used (e.g., implemented, planned to be implemented, incorporated into plans and procedures, or in some other way had affected changes made within or by the unit) and if the fact that the recommendation was developed during, and presented in, the evaluation affected the potential usage (i.e., whether the interviewees believed that something similar would have been done regardless of the evaluation). The first part’s objective was to determine how much the recommendations had been used, as perceived by the interviewees, and, by extension, to assess the evaluation’s impact.

For the second part, the interviewees were asked, generally, if the recommendations were easy to understand, put into practice, beneficial, and suitable. Although these questions were initially posed as closed questions, they were complemented by open-ended follow‑up questions to allow the interviewees to elaborate on their responses. The second part’s objective was to examine how the interviewees perceived the recommendations.

The third part of the interviews was designated to questions regarding the evaluation process. During this part, the interviewees were asked to describe their experiences of the evaluation process, particularly good and bad aspects of the process, if they could contribute with their knowledge during the process, and if they learnt something new during the process. The objective of the third part was to examine how the evaluation process (i.e., the workshops) was perceived by the interviewees.

The ten interviews were initially transcribed using the speech to text function in Microsoft Word, which was followed by manually listening to the recordings and correcting errors in the output from the automatic transcription.

To analyze the transcriptions, a directed content analysis as described by Hsieh and Shannon [54] was conducted by the same author who conducted the interviews. The directed approach was chosen due to the analysis being guided by the research questions of this study and the three distinct parts of the interviews. The directed content analysis began with the pre-identification of three broad categories corresponding to the three distinct parts of the interviews. These categories were *use of the recommendations, views of the recommendations* and *views of the evaluation process.* For the first category, use of the recommendations, pre-defined codes (yes, no, do not know) were used to classify interviewees’ answers regarding whether each recommendation had been used. Additional codes (yes, no, do not know) were then applied to assess whether the evaluation influenced the fact that the recommendation had been used. This second coding was only applicable if the initial code was yes, otherwise the response was coded as not applicable. Coding was conducted manually using Microsoft Word and Excel to organize transcripts, apply codes, and summarize frequencies. Results from the coding procedure within this category will be presented both quantitatively (i.e., frequency of each code) and qualitatively (i.e., descriptions and examples of how the interviewees reasoned) with excerpts from interviews translated into English.

For the remaining two categories, *views of the recommendations* and *views of the evaluation process*, codes were developed inductively through iterative reading of the interviewees’ responses to the corresponding questions of the interviews. The codes were then sorted into sub-categories based on similarities and relations. Coding was conducted manually using Microsoft Word and Excel to organize transcripts and apply codes. For these two categories, the results will be presented qualitatively with excerpts from interviews translated into English.

To enhance the credibility of the analysis, examples of coding decisions and interpretations were discussed among all authors throughout the process. This collaborative review served to validate the findings and reduce potential bias from a single analyst.

### Ethics

The research was conducted in accordance with the ethical principles outlined in the Declaration of Helsinki. In accordance with the Swedish Act (2003:460) concerning the ethical review of research involving humans, formal approval from the Swedish Ethical Review Authority was not required.

All evaluation workshop participants provided written informed consent during an in-person preparatory meeting held prior to the start of the online workshop series. This meeting took place on the 6^th^ of March 2023 and also marked the start and end of the recruitment process for the evaluation workshops, during which all employees of the unit agreed to participate and provided written informed consent.

For the follow-up interviews, recruitment began on the 27^th^ of February 2024 with an email sent to all employees who participated in the evaluation workshops. This email marked the start of the recruitment process and included a consent form describing the study and its implications. Participants were asked to respond by the 30^th^ of April 2024, which marked the end of the recruitment period. All interview participants provided verbal informed consent prior to each interview, after the interviewer read the consent form that participants had previously received. Participants initially gave an off-record confirmation to permit recording, followed by verbal consent documented in the interview recordings. Verbal rather than written consent was obtained because the interviews were conducted entirely online.

### Use of artificial intelligence tools

In the results section, graphical reproductions of the original Swedish strategy maps, translated into English, are presented. A large language model (M365 Copilot, based on GPT-5 architecture) was used to translate statements from Swedish to English and to recreate the visual structure of the original strategy maps, including all statements and causal links. The outputs produced by the model were reviewed and verified by the authors against the original Swedish material to ensure accuracy and completeness. The figures preserve the original statements and structure but may differ slightly in layout from the strategy maps generated by the software. The large language model was not used for data generation, analysis, or interpretation.

## Results

The results section is divided into two parts. In the first part, the results from the evaluation using the strategy mapping software are presented. The second part is devoted to the presentation of the results from the directed content analysis conducted on the transcriptions of the ten interviews.

### Evaluation results

**Fig 1:**
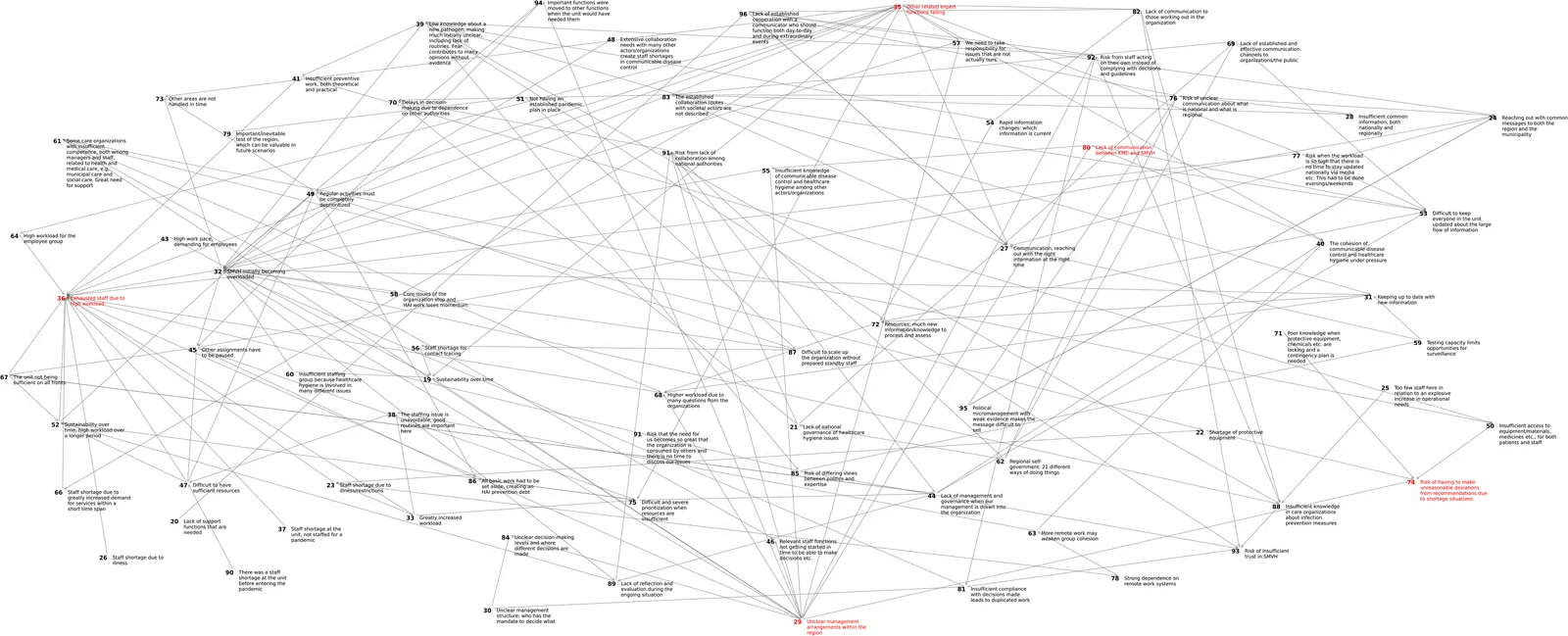
The complete risk system from the first workshop. Illustration of the application of the strategy mapping methodology displaying the risk system developed in the first workshop (red color indicates risk centrality). Adapted from the Swedish original with AI support and verified by the authors (see ‘Use of artificial intelligence tools’ in the Methods section).

**Fig 2:**
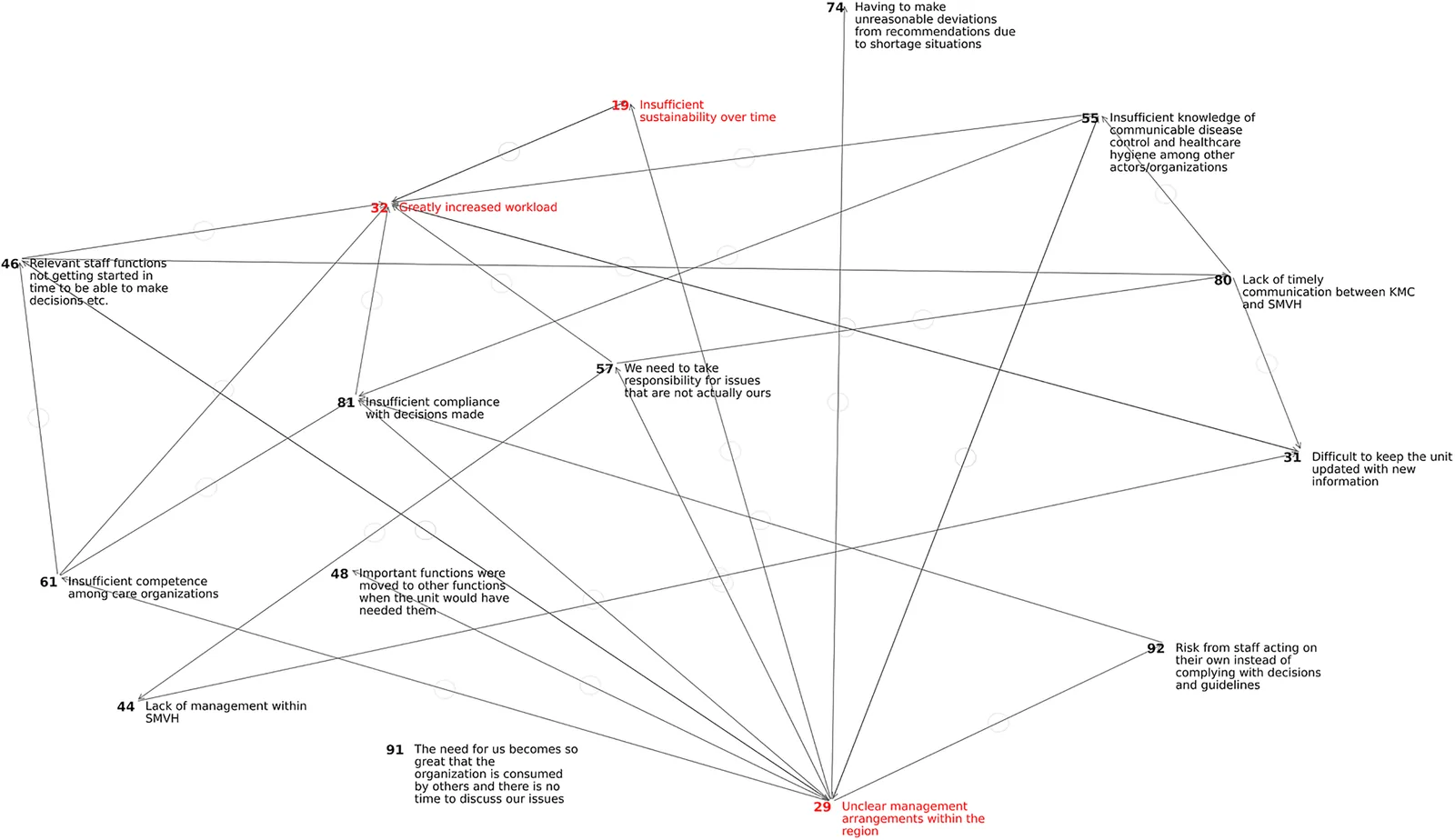
One of the subsystems from the second workshop. Illustration of the application of the strategy mapping methodology displaying a subsystem after it had been validated during the second workshop (red color indicates risk centrality). Adapted from the Swedish original with AI support and verified by the authors (see ‘Use of artificial intelligence tools’ in the Methods section).

**Fig 3:**
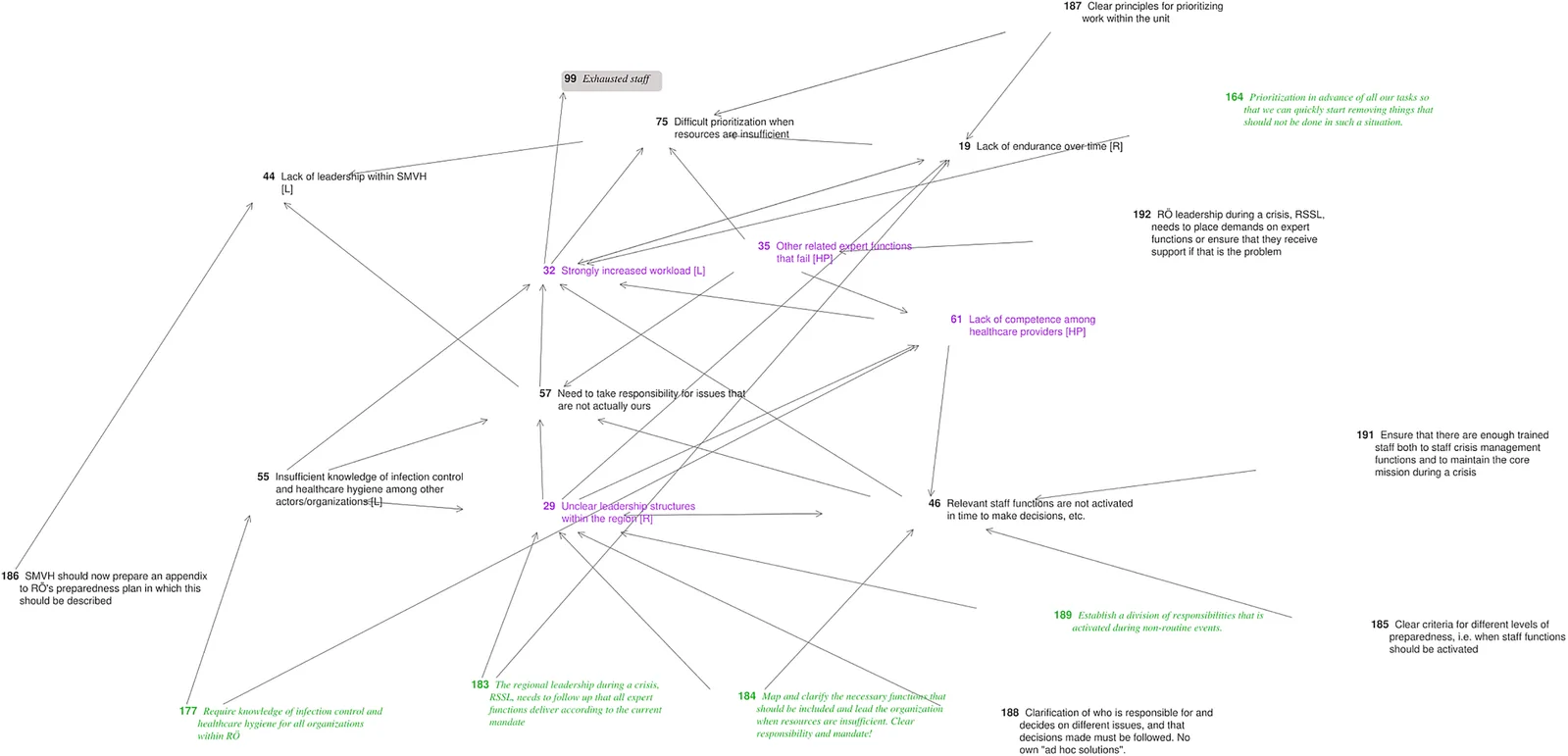
One of the subsystems from the third workshop. Illustration of the application of the strategy mapping methodology displaying a subsystem after the third workshop. The outer statements are recommendations generated by the participants (green color indicates valuable recommendations as determined by the participants (later validated during the fourth workshop), and purple color indicates potent statements). Adapted from the Swedish original with AI support and verified by the authors (see ‘Use of artificial intelligence tools’ in the Methods section).

The evaluation resulted in ten recommendations developed and assessed by the participants targeting specific potent risks as determined by the participants and the strategy mapping software’s analysis tools. The recommendations are presented in Table 2. To illustrate the application of the strategy mapping methodology and some of the progress between the workshops, Figs 1–3 are included. Fig 1 presents the complete risk system developed during the first workshop. Fig 2 presents a selected subsystem after the second workshop. Fig 3 presents a selected subsystem after the third workshop, including recommendations generated by the participants.

**Table 2:** The results (recommendations) from the evaluation, and each recommendation’s associated risk.

Recommendation	Associated risk
1) Map and define needed competences among healthcare workers related to the unit’s areas of responsibilities, and develop a plan for how these competences can be ensured.	To avoid a lack of competence among healthcare workers, something which also contributes to patient safety not being compromised.
2) Clarify which responsibilities of units within the regional public healthcare organization and other organizations within the county have for ensuring basic competence in infection control and healthcare hygiene.	To avoid the demand for the unit becoming excessive, which by extension also means that the unit will not be exposed to unreasonably high pressure and stress.
3) Identify new and effective approaches for how to educate and inform about infection control measures.	To increase the knowledge among healthcare workers in areas related to the unit’s responsibilities, something which also contributes to patient safety not being compromised.
4) Identify and describe current cooperation pathways and possible alternative cooperation pathways during crises, specific cooperation needs that may arise during crises should also be identified and described.	To ensure the use of existing and proven cooperation pathways, which by extension also helps prevent the creation of unclear guidelines.
5) Initiate regular and prioritized meetings for the entire unit in order to coordinate and share information from different ongoing projects.	To avoid ineffective communication, which by extension also means that the unit will not be exposed to unreasonably high pressure and stress.
6) Identify which and ensure how support functions that contribute to the unit’s crisis response processes can be activated.	To avoid excessive workload, which by extension also contributes to avoiding overworked staff.
7) Prioritize all the unit’s tasks so that the unit quickly can de-prioritize tasks that are not considered necessary in case of crisis.	To avoid excessive workload, which by extension also contributes to avoiding overworked staff.
8) Map and define the unit’s responsibilities during crises.	To avoid ambiguity in mandates, roles, and the unit taking responsibility of areas not within its scope, which by extension also contributes to avoiding overworked staff.
9) Map and clarify necessary functions which should oversee the unit’s operations during crises.	To avoid ambiguity in mandates and roles within the unit, which by extension also contributes to avoiding overworked staff.
10) Map and define responsibilities within the unit.	To avoid ambiguity in mandates and roles within the unit, which by extension also contributes to avoiding overworked staff.

### Content analysis

The presentation of the content analysis will be divided into three parts. First, the categorizations of the interviewees’ responses regarding each recommendation’s use, including excerpts emphasizing their reasoning, will be presented. Second, the category *views of the recommendations* will be presented together with identified sub-categories. Third, and lastly, the category *views of the evaluation process* will be presented together with identified sub-categories.

#### Use of recommendations.

For each recommendation, the interviewees were asked whether it had been used (e.g., implemented, planned to be implemented, affected changes made) and if the fact that the recommendation was developed during and presented in the evaluation affected the potential usage (i.e., whether the interviewees believed that something similar would have been done regardless of the evaluation). The coded responses to these questions from the interviewees are presented in Table 3. Following Table 3, excerpts from the interviews are presented to contextualize how the interviewees reasoned when determining the use of the recommendations.

**Table 3:** Summary of interviewees’ responses on whether each recommendation had been used and whether the evaluation affected the use.

Recommendation	Used			Use affected by the evaluation			
	Yes	No	Do not know	Yes	No	Unclear	N/A
1) Map and define needed competences among healthcare workers related to the unit’s areas of responsibilities, and develop a plan for how these competences can be ensured.	7	3	0	5	2	0	3
2) Clarify which responsibilities of units within the regional public healthcare organization and other organizations within the county have for ensuring basic competence in infection control and healthcare hygiene.	8	1	1	4	4	0	2
3) Identify new and effective approaches for how to educate and inform about infection control measures.	7	3	0	6	0	1	3
4) Identify and describe current cooperation pathways and possible alternative cooperation pathways during crises, specific cooperation needs that may arise during crises should also be identified and described.	8	0	2	5	1	2	2
5) Initiate regular and prioritized meetings for the entire unit in order to coordinate and share information from different ongoing projects.	9	1	0	6	0	3	1
6) Identify which and ensure how support functions that contribute to the unit’s crisis response processes can be activated.	3	2	5	2	0	1	7
7) Prioritize all the unit’s tasks so that the unit quickly can de-prioritize tasks that are not considered necessary in case of crisis.	5	3	2	5	0	0	5
8) Map and define the unit’s responsibilities during crises.	3	3	4	2	0	1	7
9) Map and clarify necessary functions which should oversee the unit’s operations during crises.	4	2	4	3	0	1	6
10) Map and define responsibilities within the unit.	6	1	3	2	2	2	4

As can be seen in Table 3, views varied between the interviewees for most of the recommendations regarding whether they had been used and whether the fact that the recommendations were developed in the evaluation contributed to the use. The answers provided by the interviewees can be used to understand how they reasoned as they determined the use and to contextualize why the views sometimes varied.

When participants stated that a recommendation had been used, they often referred to it as a process which had begun, rather than a complete implementation. This was, for example, referred to in relation to the first recommendation regarding mapping and defining needed competences. Furthermore, some recommendations were straightforward for the participants to determine whether they had been used due to the nature of the recommendation clearly stating an activity. One such example is the fifth recommendation regarding initiating regular meetings for the unit.

Yes, I would say that it absolutely has been started. I would not, however, say that the mapping is completely done. (Interviewee 2; referring to recommendation 1)

On Mondays, we have allocated a longer time slot for a meeting where we can prioritize together. (Interviewee 6; referring to recommendation 5)

On the other hand, when participants stated that a recommendation had not been used, they often mentioned that although the recommendation had been discussed, it had not been clearly incorporated into any ongoing projects. This was, for example, referred to in relation to the ninth recommendation, regarding the unit’s internal management. Furthermore, for some recommendations determined to not have been used, the participants referred to the recommendations as unnecessary or unspecific. One such example can be found in one participant’s reasoning when discussing the eighth recommendation, regarding defining the unit’s responsibilities during crises. This participant mentioned that not much could be done regarding defining responsibilities since it was already defined in laws and regulations.

We do not have any ongoing work related to this, but we are discussing it to some degree – what are appropriate tasks for physicians in our unit compared to tasks for nurses, for example. (Interviewee 10; referring to recommendation 9)

I think it has been defined as far as possible based on laws and regulations and so on. (Interviewee 1; referring to recommendation 8)

When reflecting on whether the evaluation affected the use of a recommendation (i.e., whether something similar to the recommendation had or would have happened disregarding the evaluation), the participants rarely mentioned that the evaluation was the sole contributing factor. However, it was common to refer to the evaluation as a contributing factor which helped emphasize what needed to be done, and thereby affecting changes made within and by the unit. On the other hand, when participants deemed that the evaluation had not affected the use of a recommendation, they often stated that the activity described by the recommendation had already been incorporated into ongoing projects before the evaluation.

Sometimes you need a structured process to emphasize those things that have been known or that have been discussed. (Interviewee 4; referring to recommendation 1)

But it was also an ongoing process even before this evaluation (Interviewee 6; referring to recommendation 2)

#### Views of the recommendations.

For the category *views of the recommendations*, four sub-categories were identified, which describe how the interviewees viewed the recommendations. These sub-categories are *low relevance to future crises and in need of continuous work, helpful, easy to understand and timely, need for clarification, too vague, similar suggestions and dependencies,* and *confirms already ongoing work.* These sub-categories will be described and exemplified through excerpts from the interviews beneath separate subheadings.

***Low relevance to future crises and in need of continuous work:*** Among the negative aspects of the recommendations, it was frequently mentioned that they could potentially be of limited relevance to future crises due to being too COVID-19 specific. Furthermore, while being too specific in what kind of situations the recommendations were developed for, it was also mentioned that the recommendations were too vague in specifying how they would affect specific functions during crises.

You know, I understand them, absolutely, but it is not always easy to, well the world changes. So, you can have a plan regarding who to contact, but then there are reorganizations and so on. (Interviewee 5)

What does this mean for my function in the unit if something happens at a school or if there is an attack or something? (Interviewee 2)

Even if the recommendations were sometimes deemed as having low relevance to future crises, some mentioned that the recommendations could easily be translated to different contexts due to their broad scope. However, such translations would require some efforts, implying that the recommendations were not ready to be used straight out of the evaluation report. Nonetheless, some interviewees stayed positive to the recommendations’ broadness despite their pandemic specificness, emphasizing that translating the recommendations to different contexts could be done with relatively low effort.

But we evaluate a pandemic and identify a lot of recommendations related to that and in that context, a context which we have not lived in for like two to three years, so it is not relevant. However, if we translate our experiences from the pandemic to things we see now, then we have things we can add to our plans based on the current situation. (Interviewee 10)

Furthermore, you might have to modify them according to the situation you are currently in. (Interviewee 8)

***Helpful, easy to understand, and timely:*** Most interviewees mentioned that, generally, the recommendations were helpful, easy to understand, and timely. In particular, the recommendations were said to provide a good basis when initiating new projects aimed at strengthening future pandemic preparedness. Some interviewees also believed that the recommendations, generally, were a major reason organizational structures had changed for the better.

The recommendations pointed out that we must create a stable ground, which I think we have done. (Interviewee 9)

Some recommendations have been really helpful, I would say. They can partly explain why we have a different structure today compared to what we have had. (Interviewee 1)

The recommendations were also discussed in relation to how the COVID-19 pandemic management would have changed if the recommendations had been presented and used before the pandemic. Related to this, it was mentioned the recommendations would have made things easier during the pandemic, something which emphasizes the usefulness of the recommendations. Furthermore, the interviewees also emphasized that the recommendations had been used, particularly by incorporating them into already planned projects and organizational changes, something which emphasizes the timeliness of the evaluation and the subsequent recommendations.

If they [the recommendations] had been in place when the pandemic came, we would have had it easier during the pandemic. (Interviewee 7)

After the pandemic we have started projects and made organizational changes to identify who we are after the pandemic and what we have learnt and how to work, and these [the recommendations] have been a part of that process. (Interviewee 10)

***Need for clarification and dependencies:*** In contrast to opinions stating that most recommendations were helpful and easy to understand, it was also frequently mentioned that the recommendations could have been improved in how they were formulated in terms of clarity. The unclarity of the recommendations was, for example, expressed by a concern that new employees who could potentially be involved in implementing the recommendations would not be able to understand them. Related to clarity, it was also mentioned that the recommendations were sometimes perceived as not being able to act upon due to being unspecific.

Perhaps it will not be that easy if you come in here and have not worked here before. Then you tell them what those things really mean, what we are supposed to do. (Interviewee 3)

I am having a hard time seeing what the new suggestions are. We have talked about education and talked about infection control measures; we always have. I would like to know, what is new? (Interviewee 2)

Additionally, and related to the clarity of the recommendations, some interviewees expressed that they were concerned that it was unclear how the unit could use the recommendations. This concern was based on the fact that the recommendations were characterized by dependencies to other units within the regional public healthcare organization or other organizations in the county. Some recommendations were therefore deemed as easy to understand, but difficult to use due to being outside the unit’s control.

They are dependent on plenty of different units and organizations, which make them difficult to use because we would need to cooperate with so many organizations just to get started. And it is by no means certain that they agree with these recommendations. (Interviewee 6)

***Confirms already ongoing work:*** According to some interviewees, one of the main advantages of the recommendations was that they emphasized areas that had been discussed previously but not prioritized. Therefore, the recommendations were mostly not seen as new suggestions, but rather as a way of prioritizing and structuring ideas and thoughts which had already been discussed informally.

I think it is also a way to highlight certain things which we had talked about before, but starting to prioritize and working. (Interviewee 4)

Advantageous in the sense that they emphasized shortcomings, and I think what we have been helped with is to understand that we need the structure. (Interviewee 9)

Since the recommendations, according to some participants, mostly confirmed already ongoing work, it was mentioned that the recommendations are not solely responsible for guiding the unit in the right direction regarding what had to be done based on experiences from the pandemic. However, it was repeatedly mentioned that the recommendations had helped and contributed to changes made, but views differed in terms of how much that could be attributed to the recommendations.

I like the recommendations, but I do not think we can praise them as the only thing which showed us what to do … but I do think that getting them presented in the report have been helpful. (Interviewee 8)

#### Views of the evaluation process.

For the category *views of the evaluation process*, five sub-categories were identified, which describe how the interviewees view the evaluation process. These sub-categories are *innovative and useful method, positive and negative aspects of the number of sessions, from the individual to the collective, too general and unspecific conclusions,* and *structured reflection and learning.* These sub-categories will be described and exemplified through excerpts from the interviews beneath separate subheadings.

***Innovative and useful method:*** When talking about the evaluation process, the interviewees frequently expressed that the employed method and software was innovative, useful, and a novel approach to evaluation which differed from traditional evaluations they had experienced. The main feature mentioned as innovative and useful was the mapping tools, which enabled a structured view of risks, shortcomings, and areas needing improvement. The usefulness of the method was also expressed through positive attitudes towards the results of the method (i.e., the recommendations).

I think that when we drew these arrows, and it resulted in this where you could group all the data which we just threw out which somehow became logical and represented the pandemic. (Interviewee 9)

I am satisfied. Even more satisfied now with the result than I was a year ago. (Interviewee 8)

***Positive and negative aspects of the number of sessions:*** The evaluation process was divided into four sessions, something which the interviewees believed to be both positive and negative. One negative aspect which was experienced during the evaluation process was the difficulty of finding the time to attend all sessions, something which caused some participants to miss occasional sessions. According to some, missing a session meant they would have difficulties during the following session due to not fully understanding the results from it. However, it was also mentioned that attending a session after missing a previous session was not associated with any difficulties due to the simplicity of the method.

I lost a bit of tempo so to speak, I thought it was difficult to be a part of the next session. (Interviewee 1)

I thought it would be difficult, but it was not. I got back into it pretty fast, and I think there was some kind of summary from the last session. (Interviewee 3)

The number of sessions also meant that the evaluation process became a somewhat drawn-out process. This caused some interviewees to feel that the evaluation process progressed too slow, and thereby decreasing their motivation to contribute throughout the evaluation process. On the other hand, it was also mentioned that the drawn-out process was much needed since it meant more time was available for reflection and quality assurance of the results.

Sometimes I felt as if it progressed too slowly and that I did not really remember all the steps. (Interviewee 8)

It has to take its time, so that we have time to create a good end product. (Interviewee 9)

***Individual and collective reflection and learning:*** One aspect of the evaluation approach which was specifically mentioned as advantageous was that the process allowed for both individual and collective reflection and learning. In particular, it was mentioned that it was positive that the first step of the process was to let each participant individually write their thoughts, which was followed by discussing and grouping the individually written thoughts collectively. This combination also allowed each participant to freely contribute based on their experiences without being influenced by the other participants.

Yes, I think it was superb because when you read someone else’s written thoughts you will get influenced by those, even if you thought of something completely different. (Interviewee 8)

I think it felt as if everybody got to have their say. (Interviewee 2)

Thanks to the individual and collective reflections, and the structured process, the interviewees mentioned that the process itself contributed to an increased understanding of the pandemic, particularly through further insights into how the pandemic was perceived differently within the unit. A significant advantage of the structured process was also that it led to concrete results with a consensus on what to do, compared to other discussion-based methods which often do not result in such clear recommendations.

I think this became a bit more concrete. That we got to reflect … could we have done anything differently, or what was good and bad? What phases of the pandemic? So, I think it was positive that we got to talk about that. (Interviewee 2)

We have done some kind of small evaluation within the group, and I think what we saw in that evaluation has been reinforced by this, and new aspects have been added so it has become broader, and we have been able to sort and agree on the recommendations. (Interviewee 9)

***Too general and unspecific conclusions:*** Although mostly positive opinions were expressed towards the evaluation process, some interviewees emphasized areas which can be improved during future evaluations using the same method. The areas emphasized by the interviewees particularly concerned the scope of and results from the process, where some expressed that the evaluation became too general and resulted in unspecific conclusions.

So, it came to be somewhat more general than what I do, I think something was missing to make it more hands-on. (Interviewee 7)

Additionally, it was also expressed that the process resulted in conclusions and recommendations which seemed to target areas outside the unit’s responsibilities. This could be explained by the method not clearly delimiting the scope to only concern areas which the unit could affect.

It was like, they should have done that, and they should have done that, and then some things were blamed on those because they did not start the regional medical command and control team. Sure, but maybe we could have done things differently as well, and I do not really think we got there. (Interviewee 1)

## Discussion

The discussion section is divided into four parts. First, the recommendations from the evaluation and the use of the recommendation are discussed. This is followed by a discussion of the evaluation process, including the interviewees’ perceptions of the process. The third part is devoted to discussing the results generally in terms of how the results can be used in future evaluations and studies of evaluations. Lastly, in the fourth part, the limitations of the study are discussed.

### The use of the recommendations

The results from the content analysis of the interview transcriptions, as presented in Table 3, show that a majority of the interviewees believed that most of the recommendations developed and presented in the evaluation had been used. Furthermore, interviewees, who believed that recommendations had been used, often stated that the evaluation had affected the use. Based on this, it is possible to conclude that the evaluation, designed in accordance with principles and assumptions from practical participatory evaluation [18] and using the strategy mapping methodology [45] incorporated into a software tool, has had an impact. However, the variations of the interviewees’ perceptions warrant an extended discussion of the recommendations.

The varying perceptions among the interviewees can have multiple explanations. One explanation could be that the interviewees stated that a recommendation had not been used when they had no knowledge of the status or simply had not heard any updates regarding the recommendation from management or colleagues (i.e., the interviewees said no rather than do not know). A second explanation is that the interviewees had different views of what it means for a recommendation to be implemented or used. Some interviewees might have viewed a recommendation to have been used when a decision had been made to implement it into plans and procedures in the future, while others only might have believed a recommendation to have been used when it had been incorporated into daily activities or perhaps even affected patient treatment. This explanation is supported by reasoning from implementation science and the evaluation research field, where multiple definitions of use and implementation exist [55–57]. A third explanation, related to the previous two, could be that perceptions depended on the roles of the interviewees within the evaluated organization. For example, some interviewees in roles such as managers may have had deeper insight into, or different definitions of, the use of the recommendations compared to, for example, subject matter experts. However, although not being explicitly analyzed, no obvious tendencies in the results suggest a clear pattern regarding whether views of recommendation use depended on interviewees’ roles.

As evident by the directed content analysis, the interviewees believed, at least to some degree, that the recommendations were actionable. These findings support the effectiveness of the approach in producing recommendations perceived as valid, jointly owned and credible. Three areas considered outcomes from a participatory evaluation process [25]. However, less positive views of the recommendations were also present. For example, it seems as if the recommendations to some degree confirmed already ongoing work, meaning that the evaluation perhaps did not produce new insights. According to the interviewees, this was, to some degree, an advantage since it meant that the evaluation provided evidence for ongoing projects and previous suggestions. It can, however, also be perceived as a disadvantage since resources are spent on producing an evaluation which, to some degree, does not provide new insights. Björnqvist et al. [2] reported similar findings, which, together with the findings from this study, raises an important question: Should evaluations primarily validate existing ideas or generate new insights?

Furthermore, some interviewees perceived the recommendations as unclear, unspecific, too general, and slightly irrelevant. These shortcomings can be assumed to be related to the recommendation development and selection process, which was carried out by the participants under the guidance of the facilitator (i.e., the facilitator did not make any significant changes to the recommendations or influence the process). A potential trade-off between quality and stakeholder involvement therefore might have occurred in the evaluation. Since the main justification was pragmatic (i.e., to conduct an impactful evaluation) the perceived low-quality of the recommendations could have limited the impact of the evaluation (although, as has been shown, the evaluation had an impact). This since the quality of an evaluation and its recommendations, just like stakeholder involvement, can affect the probability of it being used [3–6]. These results therefore raise questions about whether it would be advantageous for the facilitator to intervene actively in the process of developing recommendations to increase the quality. This involvement would, however, need to be non-intrusive in order to still reap the benefits of stakeholder involvement.

### The evaluation process

The evaluation conducted in this study, using the strategy mapping methodology incorporated into a software tool, was a drawn-out process of four separate workshops involving 15 employees of the selected unit. As evident in the content analysis, the interviewees saw the number of sessions as both positive and negative. Among the positive aspects were that the number of sessions meant that the participants could thoroughly discuss and reflect on perceived risks from the pandemic, causal links between risks and recommendations. Since these aspects include the main benefits of the strategy mapping methodology, support is provided for the necessity of a drawn-out process [42,45,46].

Among the negative aspects, it was expressed that the evaluation process progressed at too slow a pace, and that it was difficult to get back into the process if a session had been missed. Although not explicitly mentioned by the interviewees, these beliefs could contribute to unfavorable attitudes towards the evaluation and the recommendations developed during the evaluation process. Such attitudes could potentially negatively affect participants’ motivations to promote the use of recommendations from the evaluation and, if necessary, change their behaviors in accordance with the recommendations [3,6,58,59]. It therefore might be necessary to shorten the process by identifying the most and least important steps. This need is also supported by the fact that four separate workshops with 15 participants might be unpractical due to general time constraints, particularly if the method is to be used frequently when evaluating crisis management efforts.

Furthermore, the interviewees also had plenty of positive views of the evaluation process. Generally, this provides support for participatory evaluations in the context of crisis management efforts, which in turn supports suggestions previously put forward to increase stakeholder involvement in such evaluations [2,60]. Specifically, since the positive views were directed towards core features of the strategy mapping methodology such as the causal mapping process, support has been provided for suggestions to increase the usage of causal mapping in evaluations [52].

The interviewees also emphasized that the evaluation process resulted in individual learning through individual and collective reflection during the workshops, implying a certain degree of process use [61]. This aspect is something which has previously been highlighted as one of the main benefits of participatory evaluations in addition to increasing the probability of recommendations being used (i.e., the evaluation process in itself has an impact not related to the use or implementation of recommendations) [25,37]. It is also an important aspect to keep in mind when justifying, and assessing, participatory evaluations, since this kind of impact would not be possible to acquire in traditional evaluations with limited stakeholder involvement.

### Future evaluations and studies

Based on the results of this study, as well as the discussion and interpretation of the findings, there are several practical implications and scientific contributions, which may inform future evaluations and research on evaluations in the crisis management context.

A first contribution from this study is that it emphasizes the need to clearly define what use of a recommendation implies. The absence of a common definition before the interviews were conducted in this study might explain some of the variance of the interviewees’ responses. Defining use would also be beneficial in non-scientific contexts, for example for organizations aiming for structured and continuous follow ups of evaluations.

A second contribution of this study is that there seems to be a need for participatory evaluations of crisis management efforts to clearly define if the main purpose is to provide new insights into how to manage identified areas needing improvement or to collect, validate, and disseminate already existent ideas and suggestions. If the former purpose is chosen, there seems to be a need for a process which facilitates the generation of novel insights into how to manage identified risks or areas needing improvement. Such a process could be inspired by ideas from the forgetting fixation theory, which has aimed to describe how fixations on past ideas can be managed in creative problem-solving [62]. Future studies are, however, needed to determine suitable processes, and whether such processes result in novel insights from participatory evaluations of crisis management efforts.

A third contribution of this study is that the results imply that there might be a need for facilitator involvement in the recommendation development process to increase the quality of the recommendations. This finding contributes to the core discussion about the roles and competencies required of evaluators in participatory evaluations [17,63]. Based on the approach applied in this study and the results, this involvement can take different forms. One example would be to provide examples of well-written recommendations and guidelines for how to formulate recommendations, something which could increase their quality. However, such examples and guidelines might also prove negative since they could prevent participants from generating unique ideas and freely develop valuable recommendations [62]. Another form of involvement could be that the facilitator, after the recommendation development process, gathers the recommendations and modifies them to increase clarity, specificity, and actionability. This would, however, require a follow-up session where participants are able to verify, and perhaps suggest further changes to the recommendations after alterations have been made by the facilitator. This follow-up session’s design would be important to keep the benefits of the participatory evaluation approach. The benefits which could be at risk are mainly the participants’ sense of common ownership of the results and their trust in the process [16,36,37]. However, the advantages of an increased quality of the recommendations and the evaluation, emphasizes the need of future research to identify an approach, compatible with participatory evaluation, of how to increase facilitator involvement to improve the recommendations [3–6].

The aforementioned contributions related to generating recommendations are applicable to all participatory evaluations of crisis management efforts where stakeholders are involved in creating recommendations (i.e., not limited to the strategy mapping methodology applied in this study). The results do, however, also provide insights into possible changes to the evaluation process applied in this study, and which parts of the process that can, and perhaps should, be part of participatory evaluations of crisis management efforts not using the complete strategy mapping methodology.

A first aspect related to the evaluation process applied in this study which seems to need modification is the lengthy process of four separate workshops. If set to follow a similar procedure as the evaluation conducted in this study, a possible modification would be to shorten the evaluation process to only include two workshops. The workshops which could be disregarded are workshop number two and workshop number four. The second workshop, which aimed to validate the system of statements and causal links from the first workshop, could be replaced by the facilitator organizing and, to some degree, validating the systems on his/her own. This would mean that what is believed to be an important step (validation by the stakeholders) in the strategy mapping methodology is disregarded [21,42], but aspects explicitly mentioned by the interviewees in this study as positive, such as the initial causal mapping process and resulting causal map, would still be part of the evaluation. Similarly, the fourth workshop which mostly aimed to validate the developed recommendations from the third workshop could also be replaced by the facilitator organizing and, to some degree, validating the recommendations on his/her own. The removal of these two workshops could, however, weaken participants’ trust in the process and the resulting recommendations. Future research is therefore needed to determine the optimal number of workshops when conducting similar evaluations, in particular related to if comparable results in terms of impact and views of the evaluation process are attainable with fewer workshops.

Since the interviewees mostly had positive views of evaluation process, there does not seem to be a need for any major changes in addition to the suggested shortening of the process. However, it is interesting which, and how, aspects from the evaluation process in this study can be used when the complete strategy mapping methodology is not deemed as suitable, for example due to not having access to strategy mapping software. For example, features from the strategy mapping methodology could be implemented into post crisis or post exercise discussions and meetings [60]. These features could include simplified causal mapping processes using physical whiteboards or alternative software to create causal maps [64], if the aim is to identify areas needing improvement or to gain a deeper understanding of the exercise or crisis together with involved stakeholders. Additional features which could be incorporated are ratings by participants of risks or areas needing improvement, recommendation development by participants targeting specific risks or areas needing improvement, and ratings by participants of developed recommendations. However, applying only these features would make the evaluations less participatory due to a lower degree of involvement of stakeholders in data collection, interpretation, and analysis [38]. Future research is therefore needed to determine how to incorporate these features (e.g., ratings and recommendation development by stakeholders) into evaluations of crisis management efforts through post exercise or post crisis discussions, and if incorporating such processes enables learning among participants from the evaluation process as was the case in this study and as has been emphasized as a main benefit of participatory evaluation [37].

### Limitations

A limitation of this study is its reliance on a single case, which may limit generalizability. However, the single-case approach allowed for an in-depth exploration of a practical application of the methodology, which can serve as a valuable foundation for future studies and evaluations in similar contexts.

Furthermore, in this study the COVID-19 pandemic served as the case to test the strategy mapping methodology incorporated into a software tool. This can be seen as a limitation due to the pandemic’s scale and exceptional nature, which may have influenced the participants’ perceived need for evaluation and receptiveness to the evaluation process and its conclusions. However, despite this, there is little reason to believe that the results obtained and conclusions drawn in this study are limited to the pandemic context, as the focus has been on the evaluation process and the use of recommendations rather than on pandemic-specific lessons.

To determine the impact of the evaluation, interviews were conducted with ten of the 15 employees who participated in the evaluation workshops. Although this approach was determined to be the only viable one due to the unavailability of other forms of data to assess impact, such as structured logs of implemented changes, it comes with some limitations. First, as has been seen in the results and thoroughly discussed, views regarding the use of recommendations varied, which makes it difficult to determine the exact impact. Second, five of the workshop participants declined to participate in the interviews, which raises concerns that these participants may have held negative views of the evaluation process or the recommendations that they were unwilling to share. Third, relying on interviews also raises the concern of social desirability bias. This implies that it is possible that the interviewees provided answers according to what they perceived as being socially acceptable or desirable rather than their personal opinions [65]. This is particularly relevant as the interviews were conducted by the workshop facilitator. Fourth, to analyze the interviews, one author conducted the content analysis. This means that inter-coder reliability was not measured and that potentially important categories may not have been identified.

## Conclusion

This study sought to explore how strategy mapping methodology, incorporated into a software tool, could be used to conduct participatory evaluation of crisis management efforts, and how the evaluation process and its outcomes were perceived and used by stakeholders and the organization involved in, and affected by, the evaluation. The results showed that most of the recommendations from the evaluation had been used and that there were mostly positive views of the evaluation process. Based on this, it is possible to conclude that strategy mapping methodology is a viable approach for conducting impactful participatory evaluations of crisis management efforts. This also suggests that, generally, participatory evaluations are suitable for evaluating crisis management efforts. Furthermore, the results also showed that certain aspects of the evaluation process and the recommendations can be improved or changed, for example the clarity of recommendations and the length of the process. In this study it has been proposed that the facilitator ought to take a more active role in improving recommendations in terms of clarity and actionability. Future research is, however, needed to determine if actively involving the facilitator does not prevent the benefits of stakeholder involvement. Future studies also ought to be conducted to explore how perceived positive aspects of the evaluation conducted in this study, such as causal mapping, recommendation development, and participant rating of areas needing improvement and recommendations, can be incorporated into post crisis or post exercise discussions and debriefs with stakeholders when the complete strategy mapping methodology is not suitable.

To summarize, this study contributes valuable knowledge on promising approaches for increasing the impact of evaluations of crisis management efforts (i.e., the strategy mapping methodology). If the strategy mapping methodology is not feasible, it also highlights important aspects that can be incorporated into future participatory evaluations, such as the development and subsequent rating of recommendations. Applying these findings in practice may create greater opportunities for learning from past experiences and thereby contribute to improved preparedness for future crises.
