# Supplementary material for: Utilizing strategy mapping software for participatory evaluation of crisis management efforts: A case study
Source: PLoS One. 2026 Aug 20;21(8):e0356419. doi: 10.1371/journal.pone.0356419 (PMC13492745; doi:10.1371/journal.pone.0356419)
Supplement: Supplementary file 1 [file pone.0356419.s002.pdf]

Response to Reviewers: PONE-D-25-67832, Utilizing Strategy Mapping Software for Participatory Evaluation of Crisis Management Efforts: A Case Study PLOS One

#	**Reviewer / editor comment**	**Response**
	Comments from the editor:	
1.	Please ensure that your manuscript meets PLOS ONE's style requirements, including those for file naming.	We have carefully reviewed the style requirements of PLOS ONE and believe that our article now meets those requirements.
2.	Thank you for stating the following financial disclosure: [Parts of this research were conducted within the Systemic Pandemic Risk Management (SPRM) project, funded by the Research Council of Norway (project number 315444). Additional parts of the research were supported by Region Östergötland.].<br><br>Please state what role the funders took in the study. If the funders had no role, please state: ""The funders had no role in study design, data collection and analysis, decision to publish, or preparation of the manuscript.""<br><br>If this statement is not correct you must amend it as needed.<br><br>Please include this amended Role of Funder statement in your cover letter; we will change the online submission form on your behalf.	We have updated the cover letter so that our financial disclosure now states that the funders had no role in study design, data collection and analysis, decision to publish, or preparation of the manuscript.
3.	We note that the grant information you provided in the 'Funding Information' and 'Financial Disclosure' sections do not match.<br><br>When you resubmit, please ensure	We have reviewed the funding information and ensured that the grant details are now consistent across all sections.

	that you provide the correct grant numbers for the awards you received for your study in the 'Funding Information' section.	
4.	Thank you for stating the following in the Competing Interests section: [I have read the journal's policy, and the authors declare the following competing interests: Authors AB, COJ, EP, and PB are employed by an independent center within the same regional public healthcare organization as the unit for communicable disease and infection control that was evaluated in this study. The regional public healthcare organization also provided financial support for the study.].<br><br>Please confirm that this does not alter your adherence to all PLOS ONE policies on sharing data and materials, by including the following statement: ""This does not alter our adherence to PLOS ONE policies on sharing data and materials." (as detailed online in our guide for authors http://journals.plos.org/plosone/s/competing-interests). If there are restrictions on sharing of data and/or materials, please state these. Please note that we cannot proceed with consideration of your article until this information has been declared.<br><br>Please include your updated Competing Interests statement in your cover letter; we will change the online submission form on your behalf.	We have updated the cover letter so that it includes this information. However, there are some restrictions on data sharing, as can be seen in the response to comment #5. These restrictions are not connected to the potential competing interest. It is therefore true to state that the potential competing interest does not alter our adherence to the PLOS ONE policies.

5.	In the online submission form, you indicated that [Excerpts from the evaluation workshops and interviews that support the conclusions are included within the manuscript. Full interview transcripts and complete workshop results (in Swedish) can be provided upon request after anonymization, in accordance with the consent given by participants.]<br><br>All PLOS journals now require all data underlying the findings described in their manuscript to be freely available to other researchers, either 1. In a public repository, 2. Within the manuscript itself, or 3. Uploaded as supplementary information.<br><br>This policy applies to all data except where public deposition would breach compliance with the protocol approved by your research ethics board. If your data cannot be made publicly available for ethical or legal reasons (e.g., public availability would compromise patient privacy), please explain your reasons on resubmission and your exemption request will be escalated for approval.	We have changed our data availability statement so that it aligns with PLOS ONE requirements (and included it in the updated cover letter):<br><br>The analytical dataset (including the coding framework, illustrative excerpts from the transcripts, and traceability to the interview data) is deposited in Zenodo at DOI: https://doi.org/10.5281/zenodo.20410515. The dataset is stored with restricted access and can be shared upon reasonable requests and under conditions that ensure participant confidentiality, privacy, and data security. This restriction is in accordance with the consent provided by the participants and reflects the sensitive nature of the data, which may allow identification and include participants' views on workplace conditions. To request access, please contact the Linköping University Secure Repository at datamanagement@liu.se.
6.	Please include a caption for Table 2 in your manuscript.	We have clarified the caption for table 2 (previously it was labeled as "tabel 2").
7.	If the reviewer comments include a recommendation to cite specific previously published works, please review and evaluate these publications to determine whether they are relevant and should be cited. There is no requirement to	No comments regarding wishes to cite specific works are present.

	cite these works unless the editor has indicated otherwise.	
8.	*Comments from reviewer #1:*	
9.	The article is written according to the structure and guidelines of the journal. Please review it again.	We have made necessary changes to the structure and layout of the article according to journal guidelines as highlighted in the editor's comments.
10.	Regarding the use of the word crisis, is the word disaster not a more correct correction? This is a topic that is well known in the field of health. The word crisis is used in various and general fields.	We acknowledge and are aware that terms such as disaster or emergency can be used instead of crisis. However, we believe that crisis is more appropriate in this study, as the focus of the evaluation primarily was on risks related to organizational processes.
11.	In the introduction, the topic of evaluation and practice is mentioned. Many studies have been conducted in this field.	Thank you for this comment which emphasizes that we need to more clearly address previous research on evaluations of crisis management efforts. We have therefore added a description of how our research differs from previous research in the field by focusing on a method for increasing the impact of evaluations rather than, as is currently common, focusing on how to accurately assess performance. The added description can be found in row 51-56, which includes new references supporting the claim.
12.	There are many articles on topic-based and operational exercises in the field of crisis. These exercises and assessments are based on risks, either natural or man-made. According to some documents, Covid-19 is a biological crisis that may be based on the topic of man-made.	The topic of man-made versus natural risks or crises is beyond the scope of our study, as is the discussion on the nature of COVID-19. Instead, COVID-19 simply works as a case where the proposed strategy mapping method could be tested as a method for evaluation of crisis management efforts. We express this in the end of the introduction, and we have tried to clarify it further in row 116-118.
13.	Regarding performance evaluation regarding the way the government participates with local communities, this issue has many components and evaluations that	Unfortunately, we are quite unsure how to interpret this comment. However, we have further emphasized the necessity of the research through

	can be examined or whether it is the way you use tools and software that this issue is not well explained. There is a need to review the introduction and the necessity of the research.	revisions made in response to comment #11.
14.	Theoretical Background should be integrated in the introduction so that the statement of these issues can make your introduction more justifiable. If it is based on the journal's guidelines, this section should be used in the introduction so that the issue cannot be well explained.	Due to the length of the theoretical background, and since it does not contradict with the requirements of the journal, we believe that it is best to keep the theoretical background separate from the introduction. We have, however, clarified in the article (row 120-122) why we include a theoretical background section.
15.	2.2 Strategy Mapping should be brief and precise.	We are not entirely sure if this comment refers to if the methodology should be brief and precise when applied or if our description of the methodology should be brief and precise. If the former, we propose in the discussion that number of workshops perhaps should be reduced. If the latter, we believe that it is necessary to provide an in-depth description of the methodology due to it being applied in a new context.
16.	242 3 Methods needs to be reviewed carefully and clearly for the readers of the article.	Based on comments from the other reviewers we have made some clarifications in the method section, in particular related to how the interviews were conducted regarding how follow-up questions were used.
17.	This study did not address the type of study. This article is a multi-stage qualitative study or a combination of qualitative and quantitative or any other type of article. Please state it in the method and also in the abstract.	Thank you for this comment emphasizing that we need to clarify our type of study. We have added in the methods section (rows 267-273) that we used a multi-stage qualitative design with two separate stages. We have also added it to the abstract.
18.	The review conducted shows that it is a qualitative study that is based on the mentioned software. Its classification and classification have been done.	Unfortunately, we do not understand this comment and therefore cannot determine whether any changes are supposed to be made based on it.
19.	In the method, there is a need to write a precise summary and avoid	We have rewritten the introducing text in the methods section, now

	too many explanations. You can do it by paying careful attention. The complexity of the subject is reduced to the subject.	emphasizing what type of case study it is and that we employed a multi-stage approach. We believe this can function as a summary.
20.	Regarding the structure or process of how the interview questions are asked of people, whether your qualitative interview is semi-structured or an open interview, it should be mentioned in the method and abstract.	On row 389 it is stated that semi-structured interviews were conducted. We have added it to the abstract.
21.	Also, the number of people and their characteristics should be briefly mentioned in the abstract.	We have added the number of interviews to the abstract.
22.	Some topics should be included in the table so that the topic can be examined at a glance.	We found this comment somewhat unclear and are unsure which table or topics the reviewer is referring to. However, we have made changes to table 3 based on comments from other reviewers.
23.	The method of conducting the interviews should be stated carefully.	Beneath the heading 'Interviews' we describe the different parts of the interviews in detail. Based on this reviewer comment we are unsure what kind of information is missing. However, we have made updates based on comments from other reviewers (clarifying that follow-up questions were asked).
24.	The discussion needs to be examined more carefully and in depth based on the findings.	We have made several adjustments to the discussion section, often in accordance with comments and suggestions from the other reviewers regarding repetitions.
25.	In general, addressing experiences and using lessons learned is a very important issue, which I congratulate the authors of the article. The article or this level of deep and accurate data needs to be written more accurately using figures and tables to avoid explanations.	Thank you for acknowledging the importance of our work. While the latter part of the comment is somewhat unclear to us, we have reviewed the manuscript and made adjustments to improve clarity and presentation based on comments from all reviewers.
26.	*Comments from reviewer #2:*	

27.	1. How does the ‘hands-off’ facilitator approach influence the quality and actionability of the recommendations generated by participants?	In the article, we discuss this issue (starting on row 800). We argue that the hands-off approach might have caused some of the recommendations to be perceived as being of low quality. We therefore suggest that it might be beneficial for the facilitators to be more actively engaged in supporting the participants in generating actionable recommendations.
28.	2. The literature review should be presented though in a clearer manner how the present study differentiates from existing research and makes a step towards better understanding the research problem.	Thank you for this comment. We agree that it is necessary to more clearly state how the approach differs from similar approaches and why it is suitable in this context. We have therefore added a section to the introduction (row 77-86) where we explicitly state that strategy mapping is suitable due to being culturally sound and adhering to suggestions stating that mapping methodologies are suitable for participatory evaluation. Furthermore, we also highlight differences between the strategy mapping methodology and the similar participatory approach of ripple effects mapping.<br><br>We have also clarified how it differs from previous research on evaluations of crisis management efforts (row 53-56).
29.	3. The authors should give more emphasis on the importance of their and should also be clearer in the conclusion section. Overall, this paper is interesting and well-structured, and it is my belief that it will be appealing for the readers of the journal. Furthermore, the abstract it would be better to be one whole paragraph which includes the most important result, the innovation of the research article and what does this article study.	We have added a concluding paragraph in the conclusion sections (row 983-989) where we emphasize the importance of our results (i.e., stating that they can contribute to future increased preparedness). Small changes have also been made to the conclusion section in general to avoid repetitions. Furthermore, based on comments from another reviewer we have also stated the importance of the study in the introduction.

		We have made several changes to the abstract to make sure it clearly states the importance and novelty of our study and our findings.
30.	4. The paper should clearly address the following: What is the research question? Why is this question important and interesting? How did they approach the problem?	We have added a section in the introduction clearly stating why answering the research questions are important (i.e., so that insights can be gained regarding how evaluations' impact can be increased and that this is important since it can strengthen preparedness). This is in row 102-106.<br><br>Regarding what the research questions are, they are clearly stated in row 96-99 with a clarifying update based on this comment (clearly stating that the question is whether the evaluation was used by the evaluated organization).<br><br>How we approached the problem is described in the last paragraph of the introduction by stating that it is a case study. Due to changes made based on other reviewer comments, we have now clarified that it is an instrumental case study, something which perhaps makes our approach clearer.
31.	6. How robust is the measurement of 'impact', given that recommendation use is based on self-reported perceptions from only 10 interviews?	We agree that it is important to acknowledge the limitations of this approach. We have therefore added a paragraph in the limitations section (row 953-956) where we mention that relying on interviews has its limitations (in particular due to five of the workshop participants declining to be interviewed).
32.	7. Could the results be biased by the fact that participants were employees withing the same organization being evaluated?	We believe that participants being employed by the evaluated organization is appropriate, as they are best positioned to determine whether the recommendations have been used.
33.	8. How generalizable are the findings beyond the COVID-19 healthcare context, given that	The recommendations from the evaluation are not meant to be generalizable, instead the focus is on

	recommendations were sometimes perceived as context-specific ir vague?	the method for generating the recommendations (i.e., the strategy mapping methodology) we therefore believe that this is not an issue. We have clarified this in the introduction (row 116-118).<br><br>If the reviewer refers to generalizability of the recommendations for the studied organization, this concern is raised in the results starting on row 577.
34.	9. What is the trade-off between stakeholder participation and methodological rigor, especially when recommendations appear to confirm already ongoing work rather than generate new insights?	In the article we argue that there is a need to explore how it can be made sure that recommendations avoid simply confirming ongoing work in order to fulfil the full potential of evaluations (starting on row 871).
35.	*Comments from reviewer #3:*	
36.	1.Undefined Case Study Design<br>Problem: The manuscript lacks a clear and explicit case study methodology. It mentions "case study" (Page 10, Line 73) but does not define the case study design type (e.g., exploratory, explanatory, or descriptive). Table 1 only summarizes the workshop procedures (data collection) and does not address the overall case study design or analytical process.<br>The authors should explicitly state the case study design type in the Methods section and justify its alignment with the research questions. Specifically:<br>a. State the design type<br>b. Describe the case study process – beyond the workshop procedures, the authors should outline the logical steps of the case study<br>c. Clarify the unit of analysis – is it the infection control unit, the evaluation process itself, or the application of the methodology? This should be explicitly stated.	We have clarified that it is an instrumental case study (in the methodology section) and in the introduction (row 116), emphasizing that by instrumental we mean that the case was chosen as a context to explore the research question rather than because of a specific interest in the case.<br><br>We have further stated that it is a multi-stage study, with two separate stages (workshop followed by interviews). Lastly, we have made it clear that it was the evaluation process, including its application and subsequent use, which was analyzed in the multi-stage study.

37.	2. Insufficient Detail on Causal Mapping Process<br><br>2.1 Problem: As causal mapping is the key process of this study, its details are not sufficiently specified. The manuscript does not present the resulting causal map or how it evolved across workshops.<br>Suggestion: The authors should provide a detailed account of the causal mapping process that supports both methodological validity and replicability. Specifically, they should:<br><br>2.2 Present the final causal map (or a cleaned, readable version) as a figure, with nodes labeled and arrows clearly visible.<br>Report key structural properties in the text or a table: total number of nodes, total number of causal links, map density (links/nodes), number of causal loops, and centrality measures (in-degree and out-degree) for the top 5–10 risks.<br>Describe map evolution between Workshop 1 and Workshop 2: how many nodes/links were added, removed, or modified? Were any disagreements among participants resolved, and if so, how?<br><br>2.3 Explain how the causal structure guided recommendation development — for example, did recommendations target high-out-degree risks (root causes), high-in-degree risks (key consequences), or risks involved in loops?<br><br>2.4 Without these additions, the causal mapping process remains a "black box," and the study's core claim — that the methodology is	2.1 In response to another reviewer comment we have updated figure 1 into English, which we hope can enable a deeper understanding of the results from using the strategy mapping software.<br><br>2.2 We have added additional figures from workshop 2 and 3 to show how the strategy maps progressed. We believe this change, together with the description in the methods section, provides sufficient detail on the method. However, the statistics underlying each statement such as number of links and so on are difficult to extract and therefore not presented. Hopefully, the updated figures provide an answer to some of the reviewer's concerns. Additionally, we believe that the main focus of the article is not the exact calculations of the strategy mapping software, but rather how it was used and whether it produced results that came to be used.<br><br>2.3 In the methods section, we have emphasized that the recommendations primarily targeted risks in negative loops, drivers, and risks deemed as important by the participants (row 355-356).<br><br>2.4 We hope our adjustments and explanations in 2.2 and 2.3 provide sufficient evidence.

	viable — rests on insufficiently described evidence.	
38.	3.Lack of Evidence Triangulation<br>Problem: The main findings rely primarily on participant self-reports from interviews, lacking triangulation with other data sources (e.g., document analysis, observational records, external performance data).<br>Suggestion: Add at least one additional data source (e.g., meeting minutes, action plan documents, external audit reports) to corroborate the conclusion that the recommendations have been used. In the limitations section, further discuss the potential recall bias and social desirability bias associated with relying on a single data source (self-reports).	We agree with the reviewer that triangulation with additional data sources (e.g., documents) would strengthen the study. Unfortunately, such data are either unavailable or of insufficient quality (e.g., no proper documentation of changes made due to the evaluation exists). However, we have added this aspect as a limitation in the discussion and highlighted the potential for social desirability bias in relying on self-reports (row 956-960).
39.	4.Lack of Unique Contribution from COVID-19 Case<br>Problem: The study uses COVID-19 as a case, but the research process and findings are presented in a way that could apply to almost any crisis management context. The pandemic's unique features are not meaningfully integrated into the analysis or discussion.<br>Suggestion: Identify at least one unique feature of COVID-19 that could plausibly affect the evaluation process or outcomes (e.g., prolonged duration allowing for longitudinal reflection, high uncertainty forcing participants to think more systemically, specific strain on infection control units making capacity issues more salient).	In the end of the introduction, we state that the study does not aim to contribute to knowledge or lessons learned about or from the pandemic. This means that the reviewer is correct in stating that we present the process and findings in general terms, which is in line with our intentions.<br><br>However, we acknowledge the reviewer's point that the COVID-19 pandemic has unique characteristics that could influence the evaluation process. For example, its prolonged duration and sustained strain on the healthcare system may have enabled more longitudinal reflection and increased the perceived need for evaluation among participants.<br><br>To clarify this further, we have stated that the study is an instrumental case study (in response to previous reviewer comments) and added to the limitations section that the uniqueness of the COVID-19

		pandemic might have affected the participants (row 941-947). At the same time, we maintain that, due to the focus on the evaluation process and the use of recommendations, there are few reasons to believe that the conclusions are limited to the pandemic context.
40.	*Comments from reviewer #4:*	
41.	In their study, the authors analyze - based on a case study - how the strategy mapping methodology can be used to conduct participatory evaluation of crisis management efforts. An important aspect of the study is that the impact of strategy mapping was not only analyzed during the crisis itself, but also after approximately one year after the crisis ended, which provides an added value to the paper. The methodology is generally sound. From the methodological perspective, the reviewer only identified one point of possible improvement: the content analysis was conducted by only one author. Here it would been better to use at least two coders, maybe outside the group of the authors themselves. This would also have allowed to calculate inter-coder reliability and help to evaluate the coding scheme. The fact that an inter-coder-reliability was not measured should be mentioned in the limitations.	Thank you. We agree that the limitations of having only one author conducting the content analysis should be clearly emphasised in the limitations. We have therefore added this to the limitations section (row 960-962).
42.	Maybe you could discuss in the introduction why your approach is better than other approaches used in similar settings during the pandemic, such as "virtual focus groups".	In response to other reviewer comments, we have added a section in the introduction where we provide arguments for why our approach is relevant to test and how it differs from other approaches used in similar contexts. However, we have chosen not to include a comparison with virtual focus groups in the

		manuscript, as we consider such approaches to be conceptually different and not directly comparable to the approach employed in this study. This is because virtual focus groups seemingly targeted the challenges of conducting regular focus groups during the pandemic, whereas our approach concerns evaluating the pandemic after it had happened using a novel application of strategy mapping methodology.
43.	The product name of the strategy mapping tool is used too often. After the first introduction when you mention the product and its version, you should refer to it as the "strategy mapping tool" or "the tool". In Table 1, for example, you could write "video conferencing plus mapping tool".	We agree with the reviewer that the product name is mentioned too often. We have therefore replaced most of the Strategyfinder mentions with “the strategy mapping software”. Strategyfinder is still mentioned twice in the theory section (due to describing the particular software) and once in the method section when referring to the version used in our study.
44.	On page 18, lines 376-378, it appears that you asked "closed questions" to the interviewees, which appears a bit odd. Or were these accompanied by more open-ended questions? (which the reviewer would prefer…).	Thank you for noticing this. We have clarified that the interview questions were followed by open-ended questions to allow the interviewees to elaborate on their responses (row 405-407).
45.	In Table 3, you refer to the recommendations with numbers, which is extremely difficult to understand. You should describe the recommendations by adding one or two keywords so that they are better remembered.	We have added the complete recommendations to table 3 instead of labeling them as “#1” and so forth.
46.	On page 36, you discuss the conflicting, and somehow inconsistent conclusions that the interviewees had with regard to whether recommendations were used or not. What about the roles of the interviewees within the organisation? Could this have had an impact on the assessment?	We agree that it is possible that the interviewees’ roles within the organization might have affected their views regarding evaluation use. However, during our analysis no such tendencies were discovered (although not being an explicit part of the analysis). To emphasize this, we have added a paragraph in the discussion (row 780-786).

47.	The discussion section is a bit lengthy and repetitive, it should be shortened.	We have adjusted the discussion section to avoid unnecessary repetitions. These changes have also made the discussion section somewhat shorter.
48.	Figure 1 is hard to read, and since it is the original material from the interviews, it is in Swedish. While the reviewer does understand Swedish, it might be beneficial to translate the content in the figure into English to make it more understandable to a wider audience.	We have updated figure 1 so that it is now in English. To support the translation process and recreate the visual structure of the strategy map, a large language model (M365 Copilot, based on GPT-5 architecture) was used. We have added a sub-section in the method section describing that we used AI for this purpose (row 464-472).
49.	With regard to question 3 asked by the PLOS One journal above: This question relates to if the authors have made the underlying data of your research available. The reviewer checked "no" because it appears that the authors did not publish the full interview transcripts. However, the reviewer understand that there are limitations on what can be published in this regard because the full transcripts might make the participants identifiable. From the reviewer's perspective, this should not be an obstacle to publish the paper.	Thank you for your understanding. We have provided an answer to one of the editor comments regarding this issue, which adheres to the journal guidelines.
