# Supplementary material for: Utilizing strategy mapping software for participatory evaluation of crisis management efforts: A case study
Source: PLoS One. 2026 Aug 20;21(8):e0356419. doi: 10.1371/journal.pone.0356419 (PMC13492745; doi:10.1371/journal.pone.0356419)
Supplement: Peer review history

# Peer review: Decision Letter 0

**DOI:** 10.1371/journal.pone.0356419.r001

**Authors:** Umberto Baresi

22 May 2026

Dear Dr. Björnqvist,

Thank you for submitting your manuscript to PLOS ONE. After careful consideration, we feel that it has merit but does not fully meet PLOS ONE’s publication criteria as it currently stands. Therefore, we invite you to submit a revised version of the manuscript that addresses the points raised during the review process.

Please submit your revised manuscript by Jul 06 2026 11:59PM. If you will need more time than this to complete your revisions, please reply to this message or contact the journal office at plosone@plos.org. When you're ready to submit your revision, log on to https://www.editorialmanager.com/pone/ and select the 'Submissions Needing Revision' folder to locate your manuscript file.

- A letter that responds to each point raised by the academic editor and reviewer(s). You should upload this letter as a separate file labeled 'Response to Reviewers'.
- A marked-up copy of your manuscript that highlights changes made to the original version. You should upload this as a separate file labeled 'Revised Manuscript with Track Changes'.
- An unmarked version of your revised paper without tracked changes. You should upload this as a separate file labeled 'Manuscript'.

If you would like to make changes to your financial disclosure, please include your updated statement in your cover letter. Guidelines for resubmitting your figure files are available below the reviewer comments at the end of this letter.

If applicable, we recommend that you deposit your laboratory protocols in protocols.io to enhance the reproducibility of your results. Protocols.io assigns your protocol its own identifier (DOI) so that it can be cited independently in the future. For instructions see: https://journals.plos.org/plosone/s/submission-guidelines#loc-laboratory-protocols. Additionally, PLOS ONE offers an option for publishing peer-reviewed Lab Protocol articles, which describe protocols hosted on protocols.io. Read more information on sharing protocols at https://plos.org/protocols?utm_medium=editorial-email&utm_source=authorletters&utm_campaign=protocols.

As the corresponding author, your ORCID iD is verified in the submission system and will appear in the published article. PLOS supports the use of ORCID, and we encourage all coauthors to register for an ORCID iD and use it as well. Please encourage your coauthors to verify their ORCID iD within the submission system before final acceptance, as unverified ORCID iDs will not appear in the published article. *Only*  the individual author can complete the verification step; PLOS staff *cannot*  verify ORCID iDs on behalf of authors.

We look forward to receiving your revised manuscript.

Kind regards,

Umberto Baresi, Ph.D.

Academic Editor

PLOS One

Journal Requirements:

When submitting your revision, we need you to address these additional requirements.

1. Please ensure that your manuscript meets PLOS ONE's style requirements, including those for file naming. The PLOS ONE style templates can be found at

https://journals.plos.org/plosone/s/file?id=wjVg/PLOSOne_formatting_sample_main_body.pdf and

https://journals.plos.org/plosone/s/file?id=ba62/PLOSOne_formatting_sample_title_authors_affiliations.pdf

2. Thank you for stating the following financial disclosure: [Parts of this research were conducted within the Systemic Pandemic Risk Management (SPRM) project, funded by the Research Council of Norway (project number 315444). Additional parts of the research were supported by Region Östergötland.].

Please state what role the funders took in the study.  If the funders had no role, please state: ""The funders had no role in study design, data collection and analysis, decision to publish, or preparation of the manuscript.""

If this statement is not correct you must amend it as needed.

Please include this amended Role of Funder statement in your cover letter; we will change the online submission form on your behalf.

3. We note that the grant information you provided in the ‘Funding Information’ and ‘Financial Disclosure’ sections do not match.

When you resubmit, please ensure that you provide the correct grant numbers for the awards you received for your study in the ‘Funding Information’ section.

4. Thank you for stating the following in the Competing Interests section: [I have read the journal’s policy, and the authors declare the following competing interests: Authors AB, COJ, EP, and PB are employed by an independent center within the same regional public healthcare organization as the unit for communicable disease and infection control that was evaluated in this study. The regional public healthcare organization also provided financial support for the study.].

Please confirm that this does not alter your adherence to all PLOS ONE policies on sharing data and materials, by including the following statement: ""This does not alter our adherence to  PLOS ONE policies on sharing data and materials.” (as detailed online in our guide for authors http://journals.plos.org/plosone/s/competing-interests).  If there are restrictions on sharing of data and/or materials, please state these. Please note that we cannot proceed with consideration of your article until this information has been declared.

Please include your updated Competing Interests statement in your cover letter; we will change the online submission form on your behalf.

5. In the online submission form, you indicated that [Excerpts from the evaluation workshops and interviews that support the conclusions are included within the manuscript. Full interview transcripts and complete workshop results (in Swedish) can be provided upon request after anonymization, in accordance with the consent given by participants.]

All PLOS journals now require all data underlying the findings described in their manuscript to be freely available to other researchers, either 1. In a public repository, 2. Within the manuscript itself, or 3. Uploaded as supplementary information.

This policy applies to all data except where public deposition would breach compliance with the protocol approved by your research ethics board. If your data cannot be made publicly available for ethical or legal reasons (e.g., public availability would compromise patient privacy), please explain your reasons on resubmission and your exemption request will be escalated for approval.

6. Please include a caption for Table 2 in your manuscript.

7. If the reviewer comments include a recommendation to cite specific previously published works, please review and evaluate these publications to determine whether they are relevant and should be cited. There is no requirement to cite these works unless the editor has indicated otherwise.

[Note: HTML markup is below. Please do not edit.]

Reviewers' comments:

Reviewer's Responses to Questions

**Comments to the Author**

1. Is the manuscript technically sound, and do the data support the conclusions?

Reviewer #1: Partly

Reviewer #2: Yes

Reviewer #3: Partly

Reviewer #4: Yes

**********

2. Has the statistical analysis been performed appropriately and rigorously?

Reviewer #1: No

Reviewer #2: Yes

Reviewer #3: N/A

Reviewer #4: Yes

**********

3. Have the authors made all data underlying the findings in their manuscript fully available?

The PLOS Data policy

Reviewer #1: No

Reviewer #2: No

Reviewer #3: Yes

Reviewer #4: No

**********

4. Is the manuscript presented in an intelligible fashion and written in standard English?

Reviewer #1: Yes

Reviewer #2: Yes

Reviewer #3: Yes

Reviewer #4: Yes

**********

Reviewer #1: Dear authors,

Thank you for your attention to the topic of mapping the strategy in the field of Covid-19. The following topics can be reviewed.

The article is written according to the structure and guidelines of the journal. Please review it again.

Regarding the use of the word crisis, is the word disaster not a more correct correction? This is a topic that is well known in the field of health. The word crisis is used in various and general fields.

In the introduction, the topic of evaluation and practice is mentioned. Many studies have been conducted in this field.

There are many articles on topic-based and operational exercises in the field of crisis. These exercises and assessments are based on risks, either natural or man-made. According to some documents, Covid-19 is a biological crisis that may be based on the topic of man-made.

Regarding performance evaluation regarding the way the government participates with local communities, this issue has many components and evaluations that can be examined or whether it is the way you use tools and software that this issue is not well explained. There is a need to review the introduction and the necessity of the research.

Theoretical Background should be integrated in the introduction so that the statement of these issues can make your introduction more justifiable. If it is based on the journal's guidelines, this section should be used in the introduction so that the issue cannot be well explained.

2.2 Strategy Mapping should be brief and precise.

242 3 Methods needs to be reviewed carefully and clearly for the readers of the article.

This study did not address the type of study. This article is a multi-stage qualitative study or a combination of qualitative and quantitative or any other type of article. Please state it in the method and also in the abstract.

The review conducted shows that it is a qualitative study that is based on the mentioned software. Its classification and classification have been done.

In the method, there is a need to write a precise summary and avoid too many explanations. You can do it by paying careful attention. The complexity of the subject is reduced to the subject.

Regarding the structure or process of how the interview questions are asked of people, whether your qualitative interview is semi-structured or an open interview, it should be mentioned in the method and abstract.

Also, the number of people and their characteristics should be briefly mentioned in the abstract.

Some topics should be included in the table so that the topic can be examined at a glance.

The method of conducting the interviews should be stated carefully.

The discussion needs to be examined more carefully and in depth based on the findings.

In general, addressing experiences and using lessons learned is a very important issue, which I congratulate the authors of the article. The article or this level of deep and accurate data needs to be written more accurately using figures and tables to avoid explanations.

Reviewer #2:

Major Comments:

I suggest the authors answer the following suggestions based on my guidelines to improve the research article and for publication, which are the following:

1. How does the ‘hands-off’ facilitator approach influence the quality and actionability of the recommendations generated by participants?

2. The literature review should be presented though in a clearer manner how the present study differentiates from existing research and makes a step towards better understanding the research problem.

3. The authors should give more emphasis on the importance of their and should also be clearer in the conclusion section. Overall, this paper is interesting and well-structured, and it is my belief that it will be appealing for the readers of the journal. Furthermore, the abstract it would be better to be one whole paragraph which includes the most important result, the innovation of the research article and what does this article study.

4. The paper should clearly address the following: What is the research question? Why is this question important and interesting? How did they approach the problem?

5. To what extent does the strategy mapping methodology outperform traditional evaluation methods in terms of actual implementation of recommendations?

6. How robust is the measurement of ‘impact’, given that recommendation use is based on self-reported perceptions from only 10 interviews?

7. Could the results be biased by the fact that participants were employees withing the same organization being evaluated?

8. How generalizable are the findings beyond the COVID-19 healthcare context, given that recommendations were sometimes perceived as context-specific ir vague?

9. What is the trade-off between stakeholder participation and methodological rigor, especially when recommendations appear to confirm already ongoing work rather than generate new insights?

Reviewer #3:

1.Undefined Case Study Design

Problem: The manuscript lacks a clear and explicit case study methodology. It mentions "case study" (Page 10, Line 73) but does not define the case study design type (e.g., exploratory, explanatory, or descriptive). Table 1 only summarizes the workshop procedures (data collection) and does not address the overall case study design or analytical process.

The authors should explicitly state the case study design type in the Methods section and justify its alignment with the research questions. Specifically:

a. State the design type

b. Describe the case study process – beyond the workshop procedures, the authors should outline the logical steps of the case study

c. Clarify the unit of analysis – is it the infection control unit, the evaluation process itself, or the application of the methodology? This should be explicitly stated.

2. Insufficient Detail on Causal Mapping Process

Problem: As causal mapping is the key process of this study, its details are not sufficiently specified. The manuscript does not present the resulting causal map or how it evolved across workshops.

Suggestion: The authors should provide a detailed account of the causal mapping process that supports both methodological validity and replicability. Specifically, they should:

Present the final causal map (or a cleaned, readable version) as a figure, with nodes labeled and arrows clearly visible.

Report key structural properties in the text or a table: total number of nodes, total number of causal links, map density (links/nodes), number of causal loops, and centrality measures (in-degree and out-degree) for the top 5–10 risks.

Describe map evolution between Workshop 1 and Workshop 2: how many nodes/links were added, removed, or modified? Were any disagreements among participants resolved, and if so, how?

Explain how the causal structure guided recommendation development — for example, did recommendations target high-out-degree risks (root causes), high-in-degree risks (key consequences), or risks involved in loops?

Without these additions, the causal mapping process remains a "black box," and the study's core claim — that the methodology is viable — rests on insufficiently described evidence.

3.Lack of Evidence Triangulation

Problem: The main findings rely primarily on participant self-reports from interviews, lacking triangulation with other data sources (e.g., document analysis, observational records, external performance data).

Suggestion: Add at least one additional data source (e.g., meeting minutes, action plan documents, external audit reports) to corroborate the conclusion that the recommendations have been used. In the limitations section, further discuss the potential recall bias and social desirability bias associated with relying on a single data source (self-reports).

4.Lack of Unique Contribution from COVID-19 Case

Problem: The study uses COVID-19 as a case, but the research process and findings are presented in a way that could apply to almost any crisis management context. The pandemic's unique features are not meaningfully integrated into the analysis or discussion.

Suggestion: Identify at least one unique feature of COVID-19 that could plausibly affect the evaluation process or outcomes (e.g., prolonged duration allowing for longitudinal reflection, high uncertainty forcing participants to think more systemically, specific strain on infection control units making capacity issues more salient).

Reviewer #4:

In their study, the authors analyze - based on a case study - how the strategy mapping methodology can be used to conduct participatory evaluation of crisis management efforts. An important aspect of the study is that the impact of strategy mapping was not only analyzed during the crisis itself, but also after approximately one year after the crisis ended, which provides an added value to the paper. The methodology is generally sound. From the methodological perspective, the reviewer only identified one point of possible improvement: the content analysis was conducted by only one author. Here it would been better to use at least two coders, maybe outside the group of the authors themselves. This would also have allowed to calculate inter-coder reliability and help to evaluate the coding scheme. The fact that an inter-coder-reliability was not measured should be mentioned in the limitations.

Some minor changes are also recommended with regard to the content.

Maybe you could discuss in the introduction why your approach is better than other approaches used in similar settings during the pandemic, such as "virtual focus groups".

The product name of the strategy mapping tool is used too often. After the first introduction when you mention the product and its version, you should refer to it as the "strategy mapping tool" or "the tool". In Table 1, for example, you could write "video conferencing plus mapping tool".

On page 18, lines 376-378, it appears that you asked "closed questions" to the interviewees, which appears a bit odd. Or were these accompanied by more open-ended questions? (which the reviewer would prefer...).

In Table 3, you refer to the recommendations with numbers, which is extremely difficult to understand. You should describe the recommendations by adding one or two keywords so that they are better remembered.

On page 36, you discuss the conflicting, and somehow inconsistent conclusions that the interviewees had with regard to whether recommendations were used or not. What about the roles of the interviewees within the organisation? Could this have had an impact on the assessment?

The discussion section is a bit lengthy and repetitive, it should be shortened.

Figure 1 is hard to read, and since it is the original material from the interviews, it is in Swedish. While the reviewer does understand Swedish, it might be beneficial to translate the content in the figure into English to make it more understandable to a wider audience.

With regard to question 3 asked by the PLOS One journal above: This question relates to if the authors have made the underlying data of your research available. The reviewer checked "no" because it appears that the authors did not publish the full interview transcripts. However, the reviewer understand that there are limitations on what can be published in this regard because the full transcripts might make the participants identifiable. From the reviewer's perspective, this should not be an obstacle to publish the paper.

**********

what does this mean?). If published, this will include your full peer review and any attached files.

If you choose “no”, your identity will remain anonymous but your review may still be made public.

**Do you want your identity to be public for this peer review?**  For information about this choice, including consent withdrawal, please see our Privacy Policy

Reviewer #1: No

Reviewer #2: No

Reviewer #3: **Yes:** Yani Dong

Reviewer #4: No

**********

[NOTE: If reviewer comments were submitted as an attachment file, they will be attached to this email and accessible via the submission site. Please log into your account, locate the manuscript record, and check for the action link "View Attachments". If this link does not appear, there are no attachment files.]

To ensure your figures meet our technical requirements, please review our figure guidelines: https://journals.plos.org/plosone/s/figures

You may also use PLOS’s free figure tool, NAAS, to help you prepare publication quality figures: https://journals.plos.org/plosone/s/figures#loc-tools-for-figure-preparation.

NAAS will assess whether your figures meet our technical requirements by comparing each figure against our figure specifications.

# Author comment: Author response to Decision Letter 1

**DOI:** 10.1371/journal.pone.0356419.r002

1 Jul 2026

Response to each reviewer and editor comment is found in the 'Response to Reviewers' file.

Attachment Submitted filename: Response to Reviewers.pdf

# Peer review: Decision Letter 1

**DOI:** 10.1371/journal.pone.0356419.r003

**Authors:** Laura Hannah Kelly

3 Aug 2026

Utilizing strategy mapping software for participatory evaluation of crisis management efforts: A case study

PONE-D-25-67832R1

Dear Dr. Björnqvist,

We’re pleased to inform you that your manuscript has been judged scientifically suitable for publication and will be formally accepted for publication once it meets all outstanding technical requirements.

Within one week, you’ll receive an e-mail detailing the required amendments. When these have been addressed, you’ll receive a formal acceptance letter and your manuscript will be scheduled for publication.

An invoice will be generated when your article is formally accepted. Please note, if your institution has a publishing partnership with PLOS and your article meets the relevant criteria, all or part of your publication costs will be covered. Please make sure your user information is up-to-date by logging into Editorial Manager at Editorial Manager® and clicking the ‘Update My Information' link at the top of the page. For questions related to billing, please contact billing support.

If your institution or institutions have a press office, please notify them about your upcoming paper to help maximize its impact. If they’ll be preparing press materials, please inform our press team as soon as possible -- no later than 48 hours after receiving the formal acceptance. Your manuscript will remain under strict press embargo until 2 pm Eastern Time on the date of publication. For more information, please contact onepress@plos.org.

Kind regards,

Laura Kelly, PhD

Division Editor

PLOS One

Additional Editor Comments (optional):

Reviewers' comments:

Reviewer's Responses to Questions

**Comments to the Author**

Reviewer #1: All comments have been addressed

Reviewer #2: All comments have been addressed

Reviewer #4: All comments have been addressed

**********

2. Is the manuscript technically sound, and do the data support the conclusions?

Reviewer #1: Yes

Reviewer #2: Yes

Reviewer #4: Yes

**********

3. Has the statistical analysis been performed appropriately and rigorously?

Reviewer #1: Yes

Reviewer #2: Yes

Reviewer #4: N/A

**********

4. Have the authors made all data underlying the findings in their manuscript fully available?

The PLOS Data policy

Reviewer #1: Yes

Reviewer #2: No

Reviewer #4: Yes

**********

5. Is the manuscript presented in an intelligible fashion and written in standard English?

Reviewer #1: Yes

Reviewer #2: Yes

Reviewer #4: Yes

**********

Reviewer #1: (No Response)

Reviewer #2: (No Response)

Reviewer #4: Please make sure that the resolution of the figures is good enough to be easily "readable". Besides that, I have jo further comments, my suggestions from the first round of review have been taken into consideration.

**********

what does this mean?). If published, this will include your full peer review and any attached files.

If you choose “no”, your identity will remain anonymous but your review may still be made public.

**Do you want your identity to be public for this peer review?**  For information about this choice, including consent withdrawal, please see our Privacy Policy

Reviewer #1: No

Reviewer #2: No

Reviewer #4: No

**********

# Editor report: Acceptance letter

**DOI:** 10.1371/journal.pone.0356419.r004

**Authors:** Laura Hannah Kelly

PONE-D-25-67832R1

PLOS One

Dear Dr. Björnqvist,

I'm pleased to inform you that your manuscript has been deemed suitable for publication in PLOS One. Congratulations! Your manuscript is now being handed over to our production team.

At this stage, our production department will prepare your paper for publication. This includes ensuring the following:

* All references, tables, and figures are properly cited

* All relevant supporting information is included in the manuscript submission,

* There are no issues that prevent the paper from being properly typeset

You will receive further instructions from the production team, including instructions on how to review your proof when it is ready. Please keep in mind that we are working through a large volume of accepted articles, so please give us a few days to review your paper and let you know the next and final steps.

Lastly, if your institution or institutions have a press office, please let them know about your upcoming paper now to help maximize its impact. If they'll be preparing press materials, please inform our press team within the next 48 hours. Your manuscript will remain under strict press embargo until 2 pm Eastern Time on the date of publication. For more information, please contact onepress@plos.org.

You will receive an invoice from PLOS for your publication fee after your manuscript has reached the completed accept phase. If you receive an email requesting payment before acceptance or for any other service, this may be a phishing scheme. Learn how to identify phishing emails and protect your accounts at https://explore.plos.org/phishing.

If we can help with anything else, please email us at customercare@plos.org.

Thank you for submitting your work to PLOS One and supporting open access.

Kind regards,

PLOS ONE Editorial Office Staff

on behalf of

Dr. Laura Hannah Kelly

Staff Editor

PLOS One
